# Comparative Efficacy of Exercise Modalities for Motor Function Recovery After Acute Ischemic Stroke: A Systematic Review and Bayesian Network Meta-Analysis

**DOI:** 10.3390/life16081240

**Published:** 2026-07-27

**Authors:** Ziyang Yu, Shuaiwang Huang, Xiyang Peng

**Affiliations:** College of Physical Education, Hunan Normal University, Changsha 410012, China; ziyangyu@hunnu.edu.cn (Z.Y.); 202320152962@hunnu.edu.cn (S.H.)

**Keywords:** stroke, exercise modalities, motor function recovery, activities of daily living, balance function, network meta-analysis

## Abstract

Background: Exercise rehabilitation (ER) has become an important treatment regimen during the recovery phase of acute ischemic stroke (AIS). Optimal exercise prescriptions for post-stroke motor recovery remain controversial. Methods: PubMed, Embase, the Cochrane Library, and Web of Science were systematically searched through November 2025. The Cochrane risk-of-bias (RoB 1) assessment tool was utilized to assess the risk of bias in the original studies. Results: This study included 66 trials comprising 3675 patients. For balance, whole-body tilting postural training (WTPT), unilateral strength training (UST) and robot-assisted unilateral gait training (RAUGT) showed high SUCRA rankings. Activities of daily living likely improved most with robot-assisted Tai Chi training (RATCT), home rehabilitation guidance (HRG) and robot-assisted gait training (RAGT). Fugl-Meyer Assessment (FMA) gains appeared greatest following robot-assisted hand training with electrical stimulation (RAHT + ES), neural inhibition therapy (NIT) and graded motor imagery with electrical stimulation (GMI + ES). Walking endurance seemed most responsive to visual feedback training (VFT), UST and visual aids training (VAT), while functional mobility showed greatest improvement with tilt sensor-assisted gait training with electrical stimulation (TAGT + ES), gait training (GT) and virtual reality therapy with motor imagery (VRT + MI). Conclusions: This study provides the first comprehensive evidence synthesis evaluating the comparative effectiveness of specific exercise modalities across distinct recovery domains after AIS. Rather than supporting generic interventions, the findings highlight the distinct comparative advantages of various emerging technologies and traditional practices. These results offer an evidence-based framework for clinicians to optimize post-stroke rehabilitation protocols and highlight priority areas for future research on dose–response and long-term outcomes.

## 1. Introduction

Globally, stroke represents a primary contributor to mortality and long-term disability, with incidence rising by 70% over the past three decades [1,2]. From 1990 to 2019, China witnessed a particularly striking 226.5% increase in ischemic stroke incidence [3]. Despite advances in acute care, approximately 40% of survivors remain functionally dependent six months post-stroke [4], underscoring the urgent need for effective rehabilitation strategies. Current treatment regimens for acute stroke rely heavily on reperfusion therapies, including intravenous thrombolysis and mechanical thrombectomy, which have revolutionized acute stroke management [5,6,7]. However, the effectiveness of these therapies is inherently constrained by a narrow treatment window, and they often result in limited long-term functional recovery [5]. Furthermore, their efficacy in preventing permanent neurological deficits remains significantly limited [5]. More critically, even after receiving optimal acute-phase treatment, over half of stroke survivors still suffer from persistent sensorimotor, cognitive, or speech impairments [8]. Consequently, post-stroke rehabilitation plays an indispensable role in maximizing functional independence.

Exercise interventions promote motor recovery primarily through activity-dependent neuroplasticity, where repetitive, task-specific training facilitates cortical reorganization and synaptic remodeling [9]. Different exercise modalities, whether technology-assisted or traditional mind–body practices, may stimulate these neuroplastic mechanisms in distinct ways, necessitating head-to-head comparisons to determine their specific therapeutic effects. While existing reviews have confirmed the overall benefits of exercise for post-stroke motor outcomes, the current evidence base has largely been limited to broad categorical comparisons, such as aerobic training versus resistance training or high- versus low-intensity protocols [10,11]. This creates a critical evidence gap: a lack of granular, direct comparative evidence evaluating specific, clinically distinct exercise modalities across multiple functional domains. These exercise modalities include emerging technology-assisted interventions (e.g., robotics, functional electrical stimulation, virtual reality) and traditional mind–body practices (e.g., Tai Chi, Ba Duan Jin). Due to the absence of comparative evidence, it is difficult for clinicians to identify the most efficacious interventions for specific functional deficits, such as impaired balance, reduced activities of daily living (ADL), or diminished motor control.

Traditional pairwise meta-analyses are inherently limited in comparing multiple interventions simultaneously. Network meta-analysis (NMA) overcomes this limitation by synthesizing both direct and indirect evidence, enabling the simultaneous evaluation and ranking of multiple interventions within a single analytical framework. To address the evidence gap, this NMA of randomized controlled trials (RCTs) was conducted to systematically evaluate and rank the comparative effectiveness of diverse exercise modalities for functional recovery after stroke, thereby providing robust evidence to optimize clinical decision-making.

## 2. Materials and Methods

### 2.1. Study Registration

This NMA was conducted following the Preferred Reporting Items for Systematic Reviews and Meta-Analyses for Network Meta-Analyses extension (PRISMA-NMA) guidelines [12] (Supplementary Material S1) and was prospectively registered in the International Prospective Register of Systematic Reviews (PROSPERO) (CRD42024599266).

### 2.2. Eligibility Criteria

Inclusion criteria comprised: (i) Population: studies enrolling patients with acute ischemic stroke (AIS); (ii) Intervention: studies in which intervention groups received various modalities of exercise therapy, including, but not limited to, the following broad categories: balance training, lower limb training, strength training, robot-assisted training, electrical stimulation-assisted training, traditional Chinese exercise, neuromuscular training, and visual-based training.; (iii) Comparison: studies in which control groups received daily care; (iv) Outcome: studies reporting the following scores reflecting balance function, motor ability, ADL, and walking ability in stroke survivors as outcome measures: Berg Balance Scale (BBS), Fugl-Meyer Assessment (FMA), Barthel Index (BI), 6-Minute Walk Test (6MWT), and Timed Up and Go (TUG); (v) Study design: RCTs.

Exclusion criteria comprised: (i) Unpublished conference abstracts; (ii) Duplicate publications based on the same clinical trial, with the study reporting all cases or complete outcome measures retained; (iii) Studies that compared only different intensities of the same intervention, which were difficult to be linked with others in a meta-analysis, resulting in a break in the network geometry and infeasibility of comparative analysis with other studies; (iv) Studies reporting no outcome measures reflecting motor function or balance function.

### 2.3. Data Sources and Search Strategy

PubMed, Embase, the Cochrane Library, and Web of Science databases were systematically searched through November 2025. The search utilized combinations of subject headings and free-text terms, with no restrictions on region or publication year. The search strategy is detailed in Supplementary Material S2.

### 2.4. Study Selection

The retrieved articles were imported into EndNote. After deduplication, the titles or abstracts of the non-duplicate articles were screened. Potentially eligible articles were identified, and their full texts were downloaded and reviewed to determine their final eligibility. Two researchers screened the articles independently, followed by cross-checking. If any discrepancies occurred, a third researcher was consulted for adjudication.

### 2.5. Data Extraction

Prior to data extraction, we developed a standardized electronic data extraction form. The data extracted included title, DOI, first author, publication year, study design, authors, country, patient source, interventions, sample size per group, total sample size, baseline characteristics (e.g., age, sex), treatment duration, outcome measures, randomization methods, allocation concealment, blinding, blinding of outcome measures, data completeness, selective reporting, BBS, FMA, BI, 6MWT, and TUG. Two researchers independently extracted data, followed by cross-checking. If any discrepancies occurred, a third researcher was consulted for adjudication.

### 2.6. Risk of Bias Assessment

The Cochrane risk-of-bias (RoB 1) tool was utilized to assess the risk of bias in the included original studies. This tool covers multiple dimensions, including randomization, allocation concealment, blinding, blinding of outcome measures, data completeness, and selective reporting. Each dimension was rated as low risk, high risk, or some concerns. Two researchers independently assessed the risk of bias, followed by cross-checking. If any discrepancies occurred, a third researcher was consulted for adjudication.

### 2.7. Outcomes

The outcome measures in our review included the BBS, FMA, BI, 6MWT, and TUG. Baseline and post-treatment values were extracted from the included studies, and the changes in values from baseline to post-treatment were analyzed.

### 2.8. Synthesis Methods

To ensure the validity of indirect comparisons, the transitivity assumption was evaluated. Clinical homogeneity was maintained by excluding studies that solely compared different intensities of the same exercise modality, thereby minimizing confounding effects from intensity variations. Daily care was used as the common comparator to provide a stable baseline for the network. Although the included interventions differed in their underlying mechanisms and delivery modes, they share a common therapeutic goal of facilitating motor recovery through neuroplasticity. Regarding key effect modifiers, the inclusion criteria were designed to focus on stroke survivors at comparable recovery stages and with similar baseline functional levels. This design ensured that primary clinical characteristics influencing treatment response were reasonably balanced across the studies forming the network, thereby supporting the plausibility of the transitivity assumption.

A Bayesian random-effects model was used to compare exercise interventions on BBS, FMA, BI, 6MWT, and TUG. For outcomes measured on identical scales or units across studies (i.e., BBS, BI, 6MWT, TUG), the mean difference (MD) was used. However, since the FMA was evaluated using different sub-scales (upper extremity, lower extremity, total) with varying score ranges, the standardized mean difference (SMD) was applied to ensure statistical comparability across studies. The Markov Chain Monte Carlo (MCMC) method was employed for modeling. Four Markov chains were run simultaneously. A burn-in period of 20,000 iterations was set, and modeling was completed after 50,000 simulation iterations. Convergence was assessed using the shrink factor, a key diagnostic for MCMC convergence, with 50,000 iterations performed after a burn-in of 20,000 for each outcome. The Deviance Information Criterion (DIC) was used to compare model fit and global consistency. In the presence of a closed loop within the network, node-splitting analysis was employed to assess local consistency. Additionally, the interventions were ranked based on the surface under the cumulative ranking curve (SUCRA) values. League tables were created to compare the effectiveness of various interventions. All analyses were performed in Stata 15.0 (Stata Corporation, College Station, TX) and R 4.5.2 (R Development Core Team, Vienna, http://www.R-project.org, accessed on 10 January 2026). A *p*-value < 0.05 indicated a statistically significant difference.

## 3. Results

### 3.1. Study Selection Results

**Figure 1:**
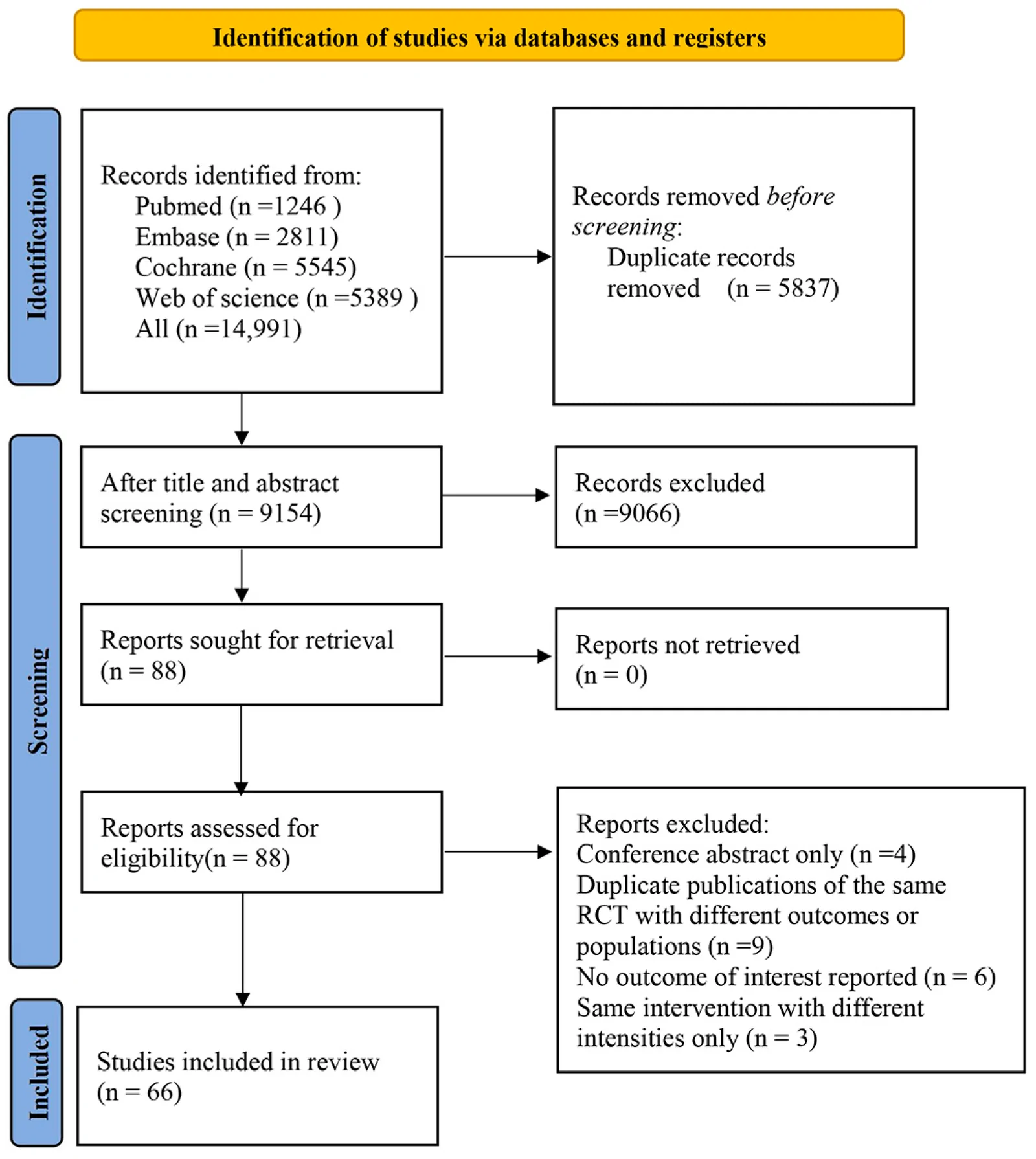
PRISMA literature screening flowchart.

We retrieved 14,991 articles from the databases and excluded 5837 duplicates. A total of 9154 articles were assessed in the title and abstract screening phase, during which 9066 articles were excluded for irrelevance to the study topic or for ineligible study designs. The full texts of 88 potentially eligible articles were downloaded and assessed. Among them, 4 were conference abstracts, 9 were duplicate publications of the same RCT based on different outcomes or populations, 6 reported no outcome of interest, and 3 were excluded because they only compared the same intervention with different intensities. Ultimately, 66 [13,14,15,16,17,18,19,20,21,22,23,24,25,26,27,28,29,30,31,32,33,34,35,36,37,38,39,40,41,42,43,44,45,46,47,48,49,50,51,52,53,54,55,56,57,58,59,60,61,62,63,64,65,66,67,68,69,70,71,72,73,74,75,76,77,78] eligible articles were selected (Figure 1).

### 3.2. Study Characteristics

The 66 included studies were published between 2008 and 2025, involving 24 countries or regions. They included 56 single-center studies and 10 multicenter studies. The total number of cases was 3675, with ages ranging from 45.4 ± 19.7 to 77.7 ± 8.9 years. Treatment durations varied from 5 days to 12 months. Among the studies, 35 reported the BBS, 29 reported the BI, 23 reported the FMA, 7 reported the 6MWT, and 12 reported the TUG (Table 1).

### 3.3. Risk of Bias in Included Studies

**Figure 2:**
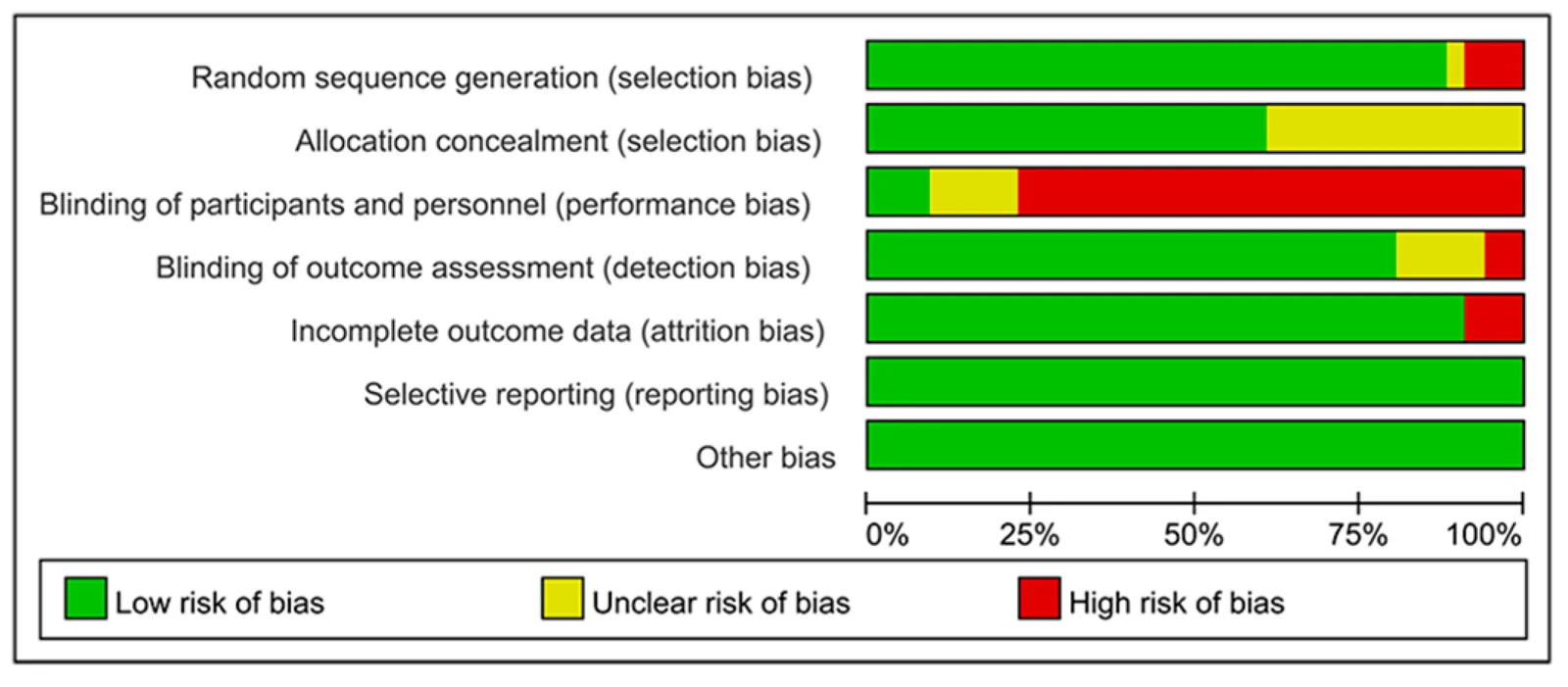
Summary of risk of bias assessment for included studies.

All the studies included were RCTs. The randomization methods adopted in these RCTs included simple randomization (primarily computer-generated numbers and random number tables) in 40 studies, block randomization in 15 studies, the envelope method in 5 studies, stratified randomization in 2 studies, and special methods in 3 studies. The remaining one did not specify the randomization method. For allocation concealment, 21 studies employed the sealed-envelope method, 5 implemented allocation concealment by computer, 3 utilized an independent third party, and 2 adopted physical methods. Another 13 studies implemented allocation concealment but did not explicitly describe the method, and the remaining 22 did not explicitly mention allocation concealment. For blinding, due to the distinct characteristics of exercise interventions, it was difficult for investigators to implement double blinding. Consequently, 5 studies employed double blinding, while 49 implemented single blinding. Among the studies with a single-blind design, the assessors were blinded in 32 studies, the raters were blinded in 7 studies, and the participants were blinded in 2 studies. The remaining 8 studies did not specify who were blinded. In addition, 12 studies did not explicitly mention blinding. Furthermore, 51 studies employed the blinding of outcome assessment (Figure 2 and Supplementary Figure S1).

The methodological limitations of the included studies directly impact the confidence in our NMA results. Specifically, 22 studies lacked explicit allocation concealment, which may introduce selection bias and inflate the estimated treatment effects. Furthermore, although the nature of exercise interventions inherently limits double-blinding, 12 studies failed to report blinding, potentially introducing detection bias for subjective or performance-based outcomes. Consequently, this potential overestimation of effects could compromise the robustness of SUCRA rankings. Therefore, while network rankings provide valuable insights, the identified optimal interventions should be interpreted with caution, acknowledging that the underlying evidence is not entirely free from bias.

### 3.4. Meta-Analysis

#### 3.4.1. Meta-Analysis of the Efficacy of Different Exercise Modalities in Increasing Berg Balance Scale Scores in Stroke Patients

**Figure 3:**
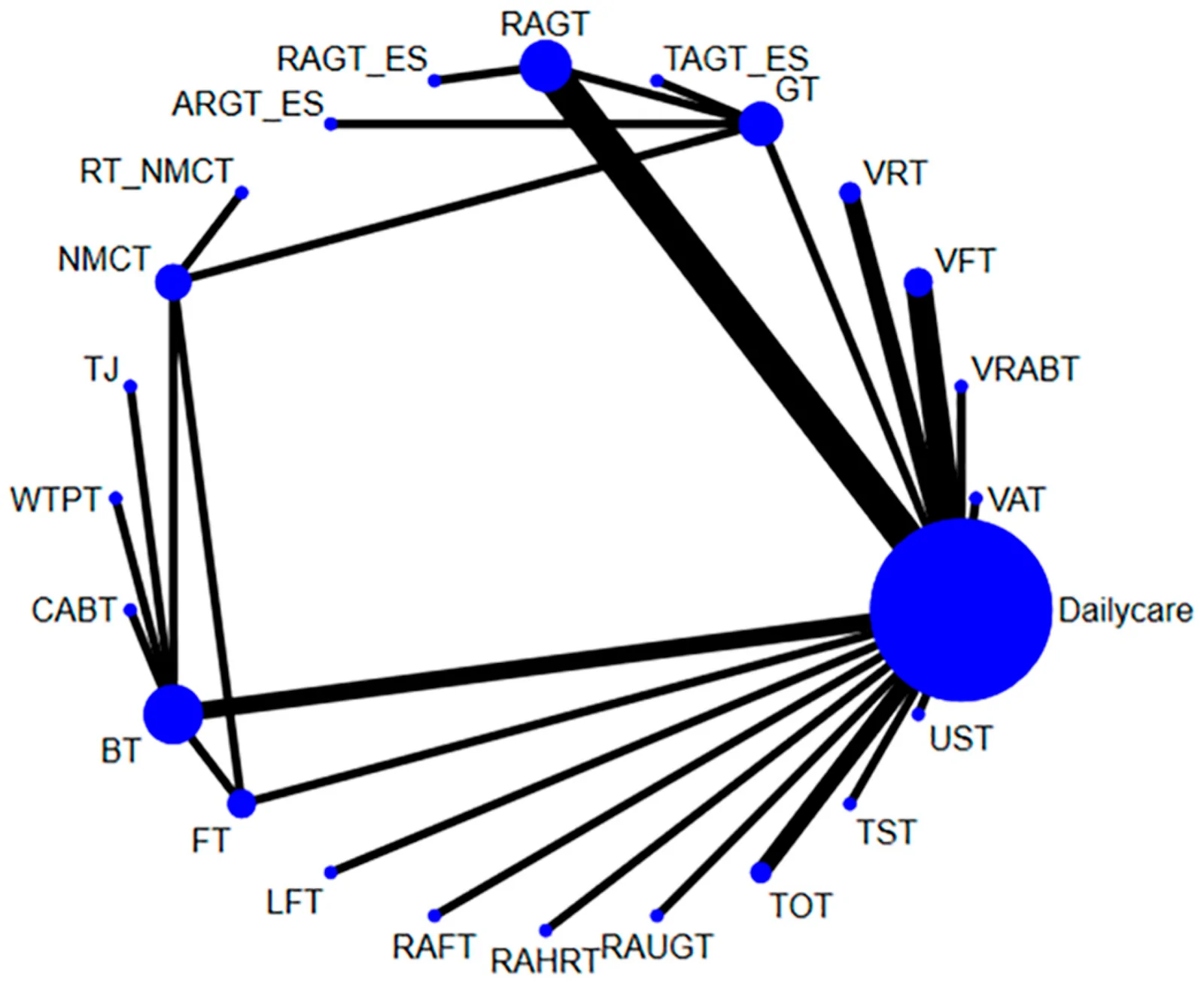
Network diagram of the efficacy of different exercise regimens on Berg Balance Scale scores during the rehabilitation phase of acute ischemic stroke. Node size is proportional to the number of participants, whereas line thickness corresponds to the number of studies directly comparing the connected interventions.

In the analysis of efficacy on BBS scores, 35 studies were included, covering 23 exercise modalities. These primarily included balance training (BT, *n* = 7), robot-assisted gait training (RAGT, *n* = 6), gait training (GT, *n* = 5), neuromuscular control training (NMCT, *n* = 4), visual feedback training (VFT, *n* = 3), functional training (FT, *n* = 3), task-oriented training (TOT, *n* = 2), and virtual reality therapy (VRT, *n* = 2). The remaining exercise regimens were included in only 1 study each. Most studies compared the interventions with daily care, though some compared different exercise modalities with one another (Figure 3). After 50,000 iterations, the shrink factor for all interventions was consistently around 1, suggesting satisfactory convergence of the model (Supplementary Figure S2a).

Compared with daily care, 12 exercise modalities significantly increased BBS scores. The top 5 were whole-body tilting postural training (WTPT) (SMD = 10.83, 95% credible interval [CrI]: 2.86–18.8), unilateral strength training (UST) (SMD = 7.48, 95% CrI: 5.28–9.7), robot-assisted unilateral gait training (RAUGT) (SMD = 10.01, 95% CrI: 0.08–20.03), augmented reality gait training (ARGT) + ES (SMD = 6.48, 95% CrI: 2.69–10.22), tilt sensor-assisted gait training (TAGT) + ES (SMD = 6.17, 95% CrI: 3.02–9.31) (Supplementary Figure S3). Notably, the wide CrIs for WTPT and RAUGT indicated statistical imprecision in their high rankings. Excluding daily care, there were a total of 41 valid positive results in the comparisons between different interventions, primarily involving UST (*n* = 11), WTPT (*n* = 7), TAGT + ES (*n* = 6), and ARGT + ES (*n* = 5). In addition to the top-ranked exercise modalities, VFT (*n* = 6), RAGT (*n* = 5), and TOT (*n* = 2) also demonstrated valid superiority in comparisons with other interventions (Supplementary Table S1). No significant publication bias was detected among the studies (Supplementary Figure S2b).

#### 3.4.2. Meta-Analysis of the Efficacy of Different Exercise Modalities in Increasing Barthel Index Scores in Stroke Patients

**Figure 4:**
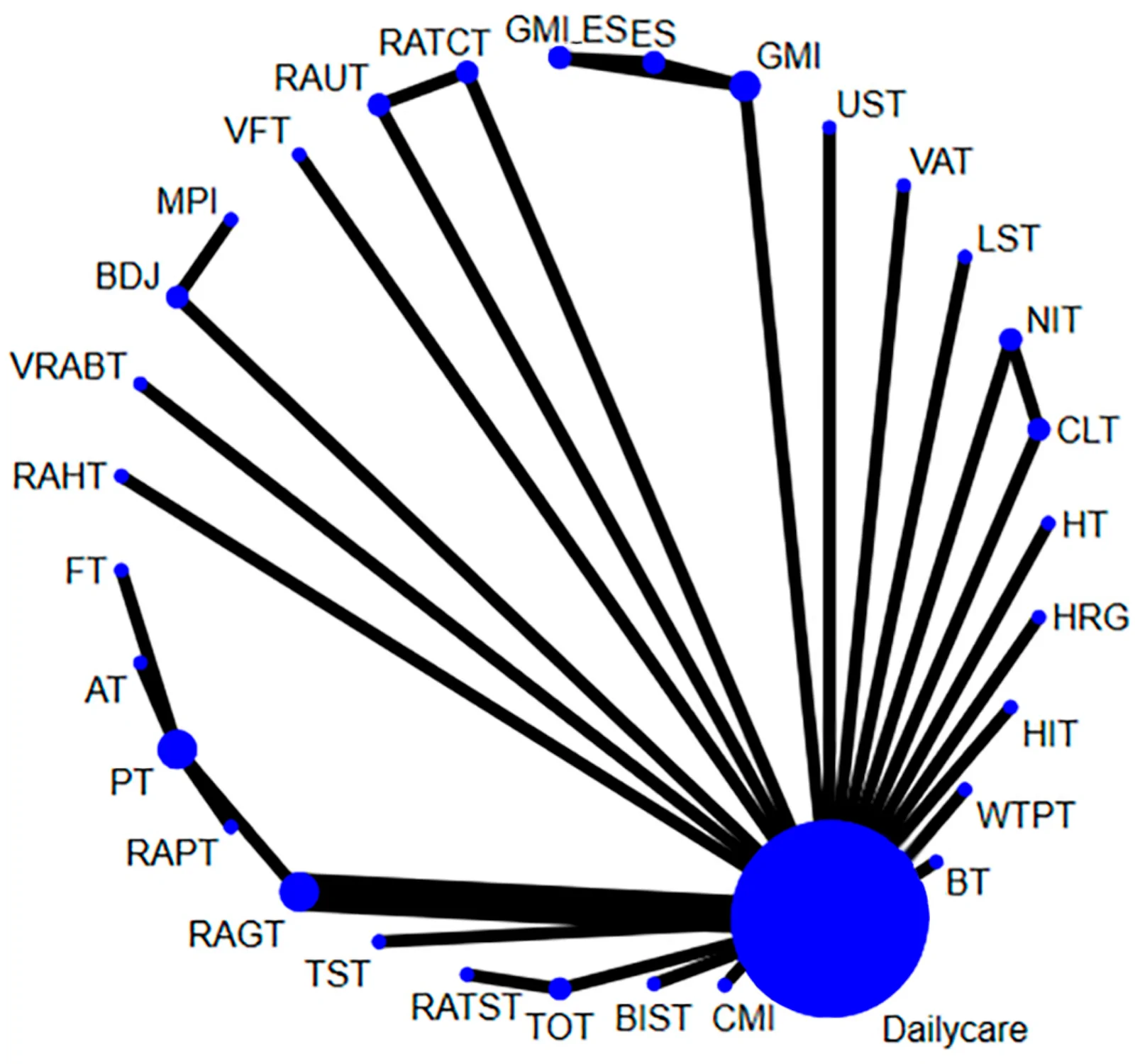
Network diagram of the efficacy of different exercise regimens on the Barthel Index scores during the rehabilitation phase of acute ischemic stroke. Node size is proportional to the number of participants, whereas line thickness corresponds to the number of studies directly comparing the connected interventions.

In the analysis of efficacy on BI scores, 29 studies were included, covering 30 exercise modalities. These primarily included RAGT (*n* = 4), physical therapy (PT, *n* = 4), BDJ (*n* = 2), TOT (*n* = 2), and BT (*n* = 2). The remaining exercise regimens were included in only 1 study each. Most studies compared the interventions with daily care, though some compared different exercise modalities with one another (Figure 4). After 50,000 iterations, the shrink factor for all interventions was consistently around 1, suggesting satisfactory convergence of the model (Supplementary Figure S4a).

Compared with daily care, 9 exercise modalities significantly increased BI scores. Based on SUCRA rankings, the leading interventions with statistical significance included robot-assisted Tai Chi training (RATCT) (SMD = 13.39, 95% CrI: 7.31–19.48), home rehabilitation guidance (HRG) (SMD = 12.44, 95% CrI: 8.41–16.45), WTPT (SMD = 10.31, 95% CrI: 0.97–19.73), robot-assisted gait training (RAGT) (SMD = 9.29, 95% CrI: 4.2–14.39), and visual feedback training (VFT) (ranked 7th, SMD = 8.43, 95% CrI: 3.39–13.5). Notably, although robot-assisted physical therapy (RAPT) ranked 3rd (SMD = 16.48, 95% CrI: −4.09–37.03) and bilateral isometric strength training (BIST) ranked 6th (SMD = 9.57, 95% CrI: −2.59–21.8) in the SUCRA ranking, their CrIs crossed zero; thus, they did not achieve statistical significance and were not counted among the 9 effective modalities (Supplementary Figure S5). Excluding daily care, there were a total of 43 valid positive results in comparisons between different interventions, primarily involving HRG (*n* = 8), RAGT (*n* = 4), robot-assisted hand training (RAHT) (*n* = 3), and TOT (*n* = 4). Medication-psychological intervention, serving as the control for comparison with BDJ, yielded 20 valid positive results in the comparisons (Supplementary Table S2). No significant publication bias was detected among the studies (Supplementary Figure S4b).

#### 3.4.3. Meta-Analysis of the Efficacy of Different Exercise Modalities in Increasing Fugl-Meyer Assessment Scores in Stroke Patients

**Figure 5:**
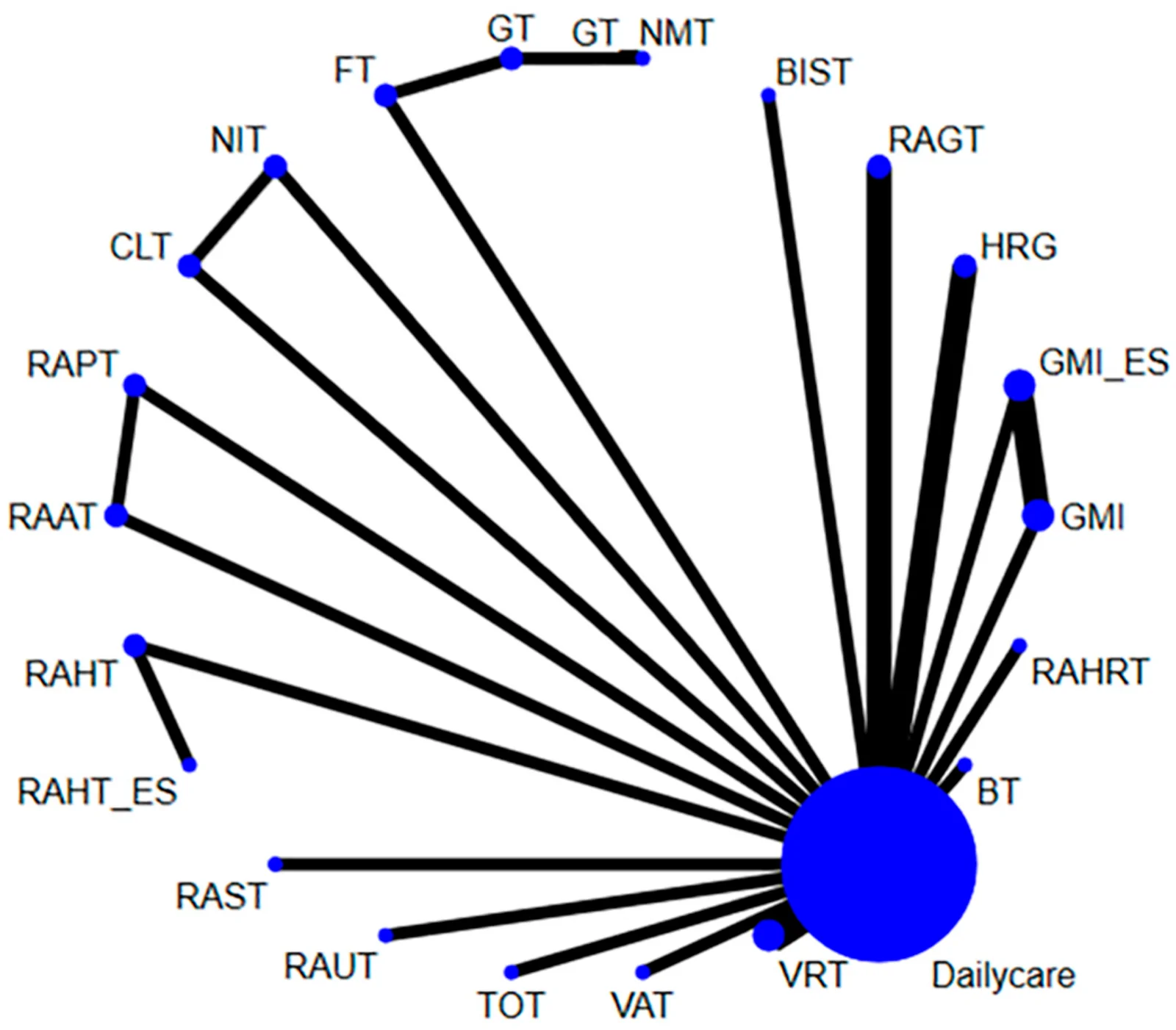
Network diagram of the efficacy of different exercise regimens on Fugl-Meyer Assessment scores during the rehabilitation phase of acute ischemic stroke. Node size is proportional to the number of participants, whereas line thickness corresponds to the number of studies directly comparing the connected interventions.

In the analysis of efficacy on FMA scores, 23 studies were included, covering 21 exercise modalities. These primarily included graded motor imagery therapy (GMI, *n* = 2), RAHT (RAHT, *n* = 2), RAGT (RAGT, *n* = 2), GT (*n* = 2), and cross-limb training (CLT, *n* = 2). The remaining exercise regimens were included in only 1 study each. Most studies compared these interventions with daily care, though some compared different exercise modalities with one another (Figure 5). After 50,000 iterations, the shrink factor for all interventions was consistently around 1, suggesting satisfactory convergence of the model (Supplementary Figure S6a).

Compared with daily care, 4 exercise modalities significantly increased FMA scores. The top 4 were robot-assisted hand training with electrical stimulation (RAHT + ES) (SMD = 1.68, 95% CrI: 0.23–3.14), neural inhibition therapy (NIT) (SMD = 0.95, 95% CrI: 0.08–1.81), graded motor imagery with electrical stimulation (GMI + ES) (SMD = 0.92, 95% CrI: 0.1–1.74), and HRG (SMD = 0.91, 95% CrI: 0.17–1.55) (Supplementary Figure S7). No significant publication bias was detected among the studies (Supplementary Figure S6b).

#### 3.4.4. Meta-Analysis of the Efficacy of Different Exercise Modalities in Increasing 6-Minute Walk Test Distance in Stroke Patients

**Figure 6:**
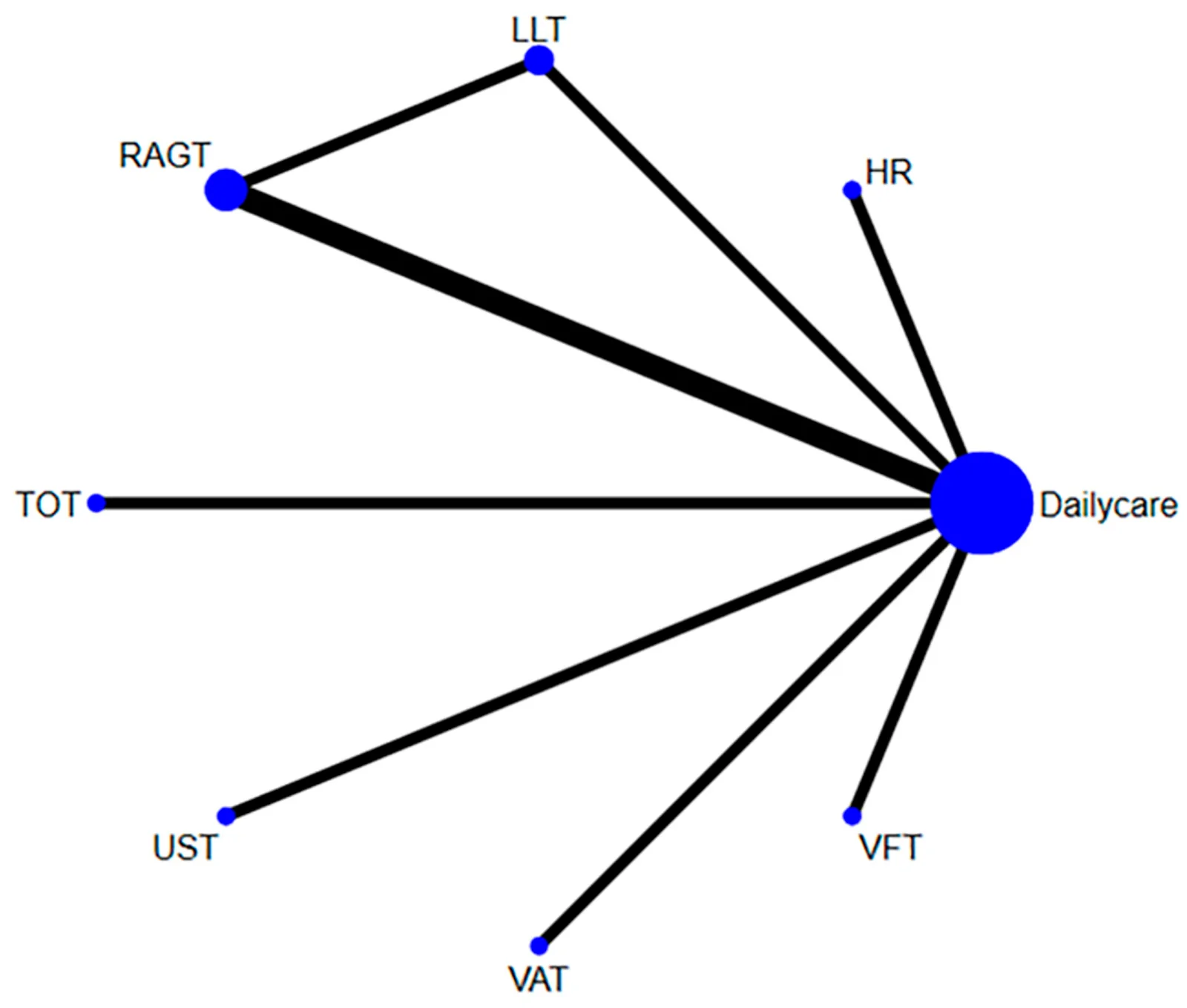
Network diagram of the efficacy of different exercise regimens on the 6-Minute Walk Test distance during the rehabilitation phase of acute ischemic stroke. Node size is proportional to the number of participants, whereas line thickness corresponds to the number of studies directly comparing the connected interventions.

In the analysis of efficacy on 6MWT distance, 7 studies were included, covering 7 exercise modalities (Figure 6). After 50,000 iterations, the shrink factor for all interventions was consistently around 1, suggesting satisfactory convergence of the model (Supplementary Figure S8a). Compared with daily care, 4 exercise modalities significantly improved the 6MWT results. These were VFT (SMD = 63.25, 95% CrI: 28.36–97.99), UST (SMD = 54.55, 95% CrI: 46.81–62.34), visual aids training (VAT) (SMD = 49.47, 95% CrI: 17.47–81.38), and RAGT (SMD = 48.67, 95% CrI: 35.49–61.75). Excluding daily care, there were 3 positive results in comparisons between different interventions, involving VFT, UST, and RAGT, respectively, all compared with lower limb training (Supplementary Figure S9). No significant publication bias was detected among the studies (Supplementary Figure S8b). Based on our overall findings, we speculated that for patients with ischemic stroke and severe neurological deficits, traditional high-intensity exercises directly targeting the lower limbs might not be optimal for improving walking ability and endurance. Instead, a progressive exercise regimen indirectly acting on the patient’s target muscle groups could be preferable. While such modalities may not act in isolation, they may influence a broader range of muscle groups, potentially facilitating the restoration of motor function (Supplementary Table S4).

#### 3.4.5. Meta-Analysis of the Efficacy of Different Exercise Modalities in Shortening Timed up and Go Test Time in Stroke Patients

**Figure 7:**
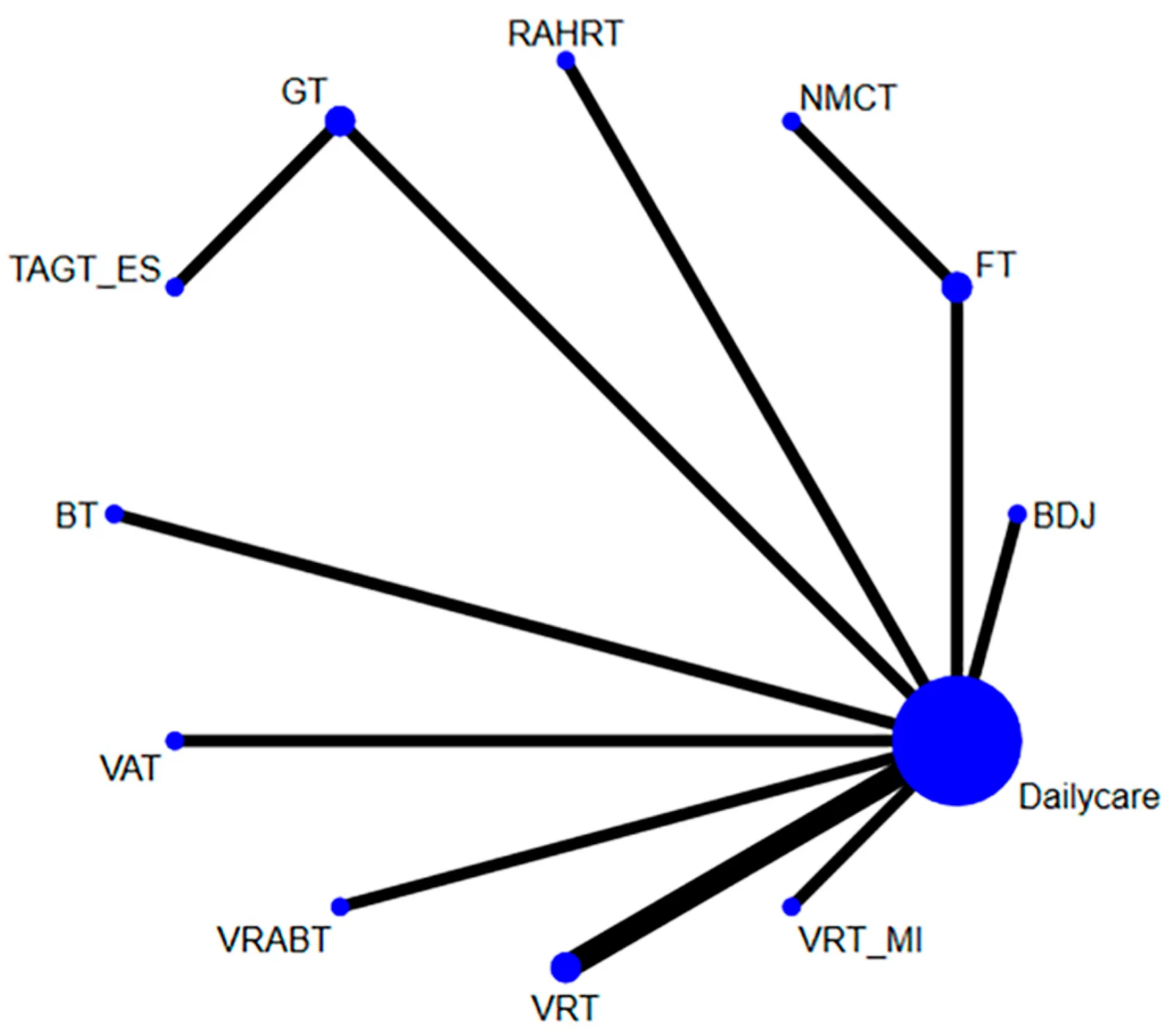
Network diagram of the efficacy of different exercise regimens on the Timed Up and Go Test time during the rehabilitation phase of acute ischemic stroke. Node size is proportional to the number of participants, whereas line thickness corresponds to the number of studies directly comparing the connected interventions.

In the analysis of efficacy on TUG time, 12 studies were included, covering 11 exercise modalities (Figure 7). After 50,000 iterations, the shrink factor for all interventions was consistently around 1, suggesting satisfactory convergence of the model (Supplementary Figure S10a). Compared with daily care, 4 exercise modalities significantly shortened the TUG time. These were TAGT + ES (SMD = 12.32, 95% CrI: 6.49–18.2), gait training (GT) (SMD = 8.93, 95% CrI: 3.61–14.3), virtual reality therapy with motor imagery (VRT + MI) (SMD = 7.38, 95% CrI: 0.63–14.09), and balance training (BT) (SMD = 1.90, 95% CrI: 0.598–3.21), even though BT ranked 8th in the SUCRA ranking. Conversely, although VAT ranked 4th in the SUCRA ranking (SMD = 10.94, 95% CrI: −18.22–40.42), its CrI crossed zero; thus, it did not achieve statistical significance and was not counted among the 4 effective modalities (Supplementary Figure S11). No significant publication bias was detected among the studies (Supplementary Figure S10b). Excluding daily care, there were a total of 10 positive results in the comparisons between different interventions, with TAGT + ES accounting for 6 of them. This multimodal exercise approach, which combined multiple assistive functions, stimulated proprioceptive responses and enhanced neuroplasticity and neural reorganization in the brain. Compared with conventional GT, this approach could significantly improve patients’ gait ability and reduce the risk of falls (Supplementary Table S5).

## 4. Discussion

### 4.1. Optimal Exercise Modality for Increasing BBS Scores

Our analysis identified WTPT, UST, and ARGT + ES as potentially the most effective interventions for increasing BBS scores. The superiority of these multimodal interventions can be explained through their shared capacity to enhance proprioceptive input and recalibrate postural control mechanisms. WTPT strategically reduces visual reliance while amplifying somatosensory stimulation from the lower extremities, thereby facilitating implicit learning of unconscious postural adjustments [51]. ARGT + ES and TAGT + ES further augment proprioceptive feedback during knee flexion and ankle dorsiflexion, which are movements critically involved in maintaining stability during the stance-to-swing transition [69].

Given that ankle dorsiflexion capacity is essential for post-stroke balance and aligns closely with the objectives of the BBS assessment, interventions that specifically target peri-ankle muscle groups through unstable surface training or functional electrical stimulation create conditions that force fine-tuning of postural responses [79,80]. Functional electrical stimulation directly activates the deep peroneal nerve to induce tibialis anterior contraction, thereby reinforcing sensorimotor integration [81]. This mechanistic explanation aligns with the high ranking of protocols that integrate sensory augmentation, such as ARGT + ES and TAGT + ES, in our NMA.

The notable efficacy of UST introduces an additional mechanistic dimension, cross-education, whereby unilateral strength gains transfer to the contralateral limb via neural adaptations at both cortical and spinal levels [82]. This phenomenon challenges the emphasis of traditional rehabilitation on bilateral symmetry and suggests that targeted training of the less-affected limb may unmask latent motor capacity in the paretic side [30]. By clarifying that modalities which specifically augment ankle proprioception and sensory integration yield the greatest benefits, our findings tentatively refined earlier systematic reviews that broadly recommended combined exercises and high-intensity training for improving post-stroke balance [83,84]. Collectively, these results raise the possibility that optimal balance recovery may depend not merely on movement repetition but on the deliberate integration of multimodal sensory cues and the strategic engagement of cross-education mechanisms.

### 4.2. Optimal Exercise Modality for Increasing BI Scores

For ADL measured by the BI scores, RATCT, HRG, and RAGT appeared to be the most effective interventions. The common mechanistic thread linking these modalities is their capacity to translate motor gains into ecologically valid, goal-directed actions through sustained engagement and contextual relevance. RATCT exemplifies this integration by combining the Cloud Hands movement of traditional Chinese Tai Chi with rehabilitation robots and animated visual feedback, an approach suitable even for early-stage patients due to its low intensity and comprehensive assistive effects [14]. HRG extends this principle into the home setting, where its core advantages lie in flexibility to adapt to individual needs, diversity of exercise options, and compatibility with multiple modalities in combination. However, the necessity of professional supervision cannot be overstated; without adequate oversight, home-based exercise may not yield optimal improvements in ADL, as highlighted in prior trials [85]. The integration of virtual reality technology into home rehabilitation provides a promising solution to this limitation by enabling remote supervision and simulated ADL practice while ensuring patient safety [42].

Robot-assisted interventions, including RAGT, RAHT, and RAPT, further contribute to ADL recovery through distinct but complementary mechanisms. RAGT provides multi-joint proprioceptive feedback via exoskeleton assistance, effectively reducing fear of falling and facilitating rhythmic motor skill integration [40]. The involvement of distal effectors, particularly wrist and hand dexterity addressed by RAHT and bilateral isometric strength training, is essential for ADL recovery because fine-grained corticospinal control of these effectors is disproportionately impaired after stroke [72]. Our findings tentatively refine earlier systematic reviews by supporting the hypothesis that optimal ADL recovery may require the deliberate embedding of functional tasks within ecologically valid contexts, supported by supervision structures, whether in-person or technology-mediated, which ensure sustained, high-quality engagement. For patients seeking to improve BI scores, home-based rehabilitation assisted by training that simulates real-life scenarios, such as virtual reality and task-oriented training, may be considered a promising option.

### 4.3. Optimal Exercise Modality for Increasing FMA Scores

For motor control assessed by the FMA, RAHT + ES, NIT, GMI + ES, and HRG seemed to demonstrate the greatest efficacy. The FMA, developed based on Brunnstrom’s sequential recovery stages, quantifies both synergistic movement patterns and isolated voluntary control [86]. This sensitivity to abnormal synergies, a hallmark of persistent neurological deficit after stroke, explains why interventions targeting synergy disruption achieve superior outcomes [86].

RAHT + ES addresses obligatory flexion synergies by immobilizing the elbow during grasping, thereby suppressing compensatory trunk motion and facilitating dissociated movement. Electrical stimulation functions by modulating spinal and cortical excitability; functional electrical stimulation directly activates peripheral nerves and, when combined with volitional movement, can restore presynaptic and long-loop inhibitory connections [81]. NIT operates through a complementary mechanism, enhancing selective motor control by targeting hyperexcitable spinal reflex arcs via reflex inhibition patterns. GMI + ES engages motor imagery to activate sensorimotor networks without requiring overt movement of the paretic limb, helping maintain cortical excitability and prevent learned nonuse during the acute phase [32].

By identifying specific active protocols and synergistic combinations of peripheral stimulation with cognitive strategies, our findings tentatively refine prior reviews that broadly endorsed task-specific training [87]. Clinically, our results support a phased approach: GMI + ES during the acute phase to preserve excitability; RAHT + ES and NIT during recovery to disrupt synergies; and HRG throughout to ensure continuity of functional gains. Collectively, optimal motor control recovery requires active patient engagement, synergy disruption, and preservation of cortical excitability.

### 4.4. Optimal Exercise Modality for Improving TUG and 6MWT Outcomes

For walking endurance measured by 6MWT, VFT and UST emerged as the most effective interventions, followed by VAT and RAGT. These findings can be understood through the physiological determinants of sustained ambulation. The 6MWT primarily reflects cardiorespiratory endurance and sustained force-generating capacity of lower-limb musculature. Accordingly, VFT, which provides real-time visual feedback on joint kinematics and weight distribution during walking, enables patients to monitor and correct gait deviations in real time, thereby improving mechanical efficiency and reducing the metabolic cost of ambulation. Studies have demonstrated that balance training with visual feedback significantly increases 6MWT distance compared to routine physical therapy alone in individuals affected by chronic stroke [54]. UST augments endurance through cross-education: strength training of the non-paretic side promotes recovery of mobility and muscle strength of the paretic side, with trials reporting significantly greater gains in 6MWT distance in the intervention group compared with the usual care group [30].

For functional mobility assessed by TUG, TAGT + ES seemed to be the optimal intervention, with GT, VRT + MI, and VAT also demonstrating significant efficacy. The TUG evaluates dynamic balance, turning agility, and rapid postural transitions, capacities that depend critically on sensorimotor integration rather than sustained force production alone. TAGT + ES addresses these deficits by combining treadmill gait training with tilt sensor-triggered electrical stimulation, which provides precisely timed afferent input during the stance-to-swing transition when ankle dorsiflexion is most critical for stability. This approach has been shown to significantly improve TUG performance and enhance the muscle architecture of the tibialis anterior on the paretic side [69].

Notably, the differential efficacy patterns observed across these two outcomes refine previous meta-analyses that broadly endorsed aerobic exercise for walking capacity [87]. Our findings extend this work by raising the possibility that endurance gains depend more heavily on strength augmentation and movement efficiency, whereas mobility improvements require targeted sensorimotor recalibration of gait phases. Collectively, these results advocate for a bifurcated clinical approach: endurance deficits warrant emphasis on visual feedback and strength training modalities, whereas mobility impairments require sensor-assisted interventions that enhance proprioceptive feedback and ankle control during postural transitions.

### 4.5. Limitations of the Study

This study has several limitations. First, although a wide variety of exercise interventions are included, the small sample sizes for some interventions may constrain the interpretation of our findings. Second, exercise intensity and monitoring standards vary across studies without standardized quantitative metrics, which makes it difficult to precisely define the optimal dose–response relationship. In addition, due to the nature of exercise interventions, it is difficult to blind both participants and intervention providers, which may introduce some implementation bias. Moreover, most studies lack medium- to long-term follow-up data, and the sustainability of the efficacy of exercise interventions needs to be further validated. Additionally, the geographical concentration of included studies (predominantly from China) may limit the generalizability of findings given regional differences in healthcare practices. Furthermore, due to the absence of a formal certainty of evidence evaluation (e.g., GRADE or CINeMA framework) the certainty of evidence for different study results cannot be quantified, which may affect the reliability of estimated intervention effectiveness. Moreover, the exclusion of studies comparing different intensities of the same intervention (*n* = 3) may introduce bias, as dose–response relationships are critical for clinical decision-making, and their absence may impact the completeness of evidence.

## 5. Conclusions

In summary, our findings demonstrate distinct comparative advantages of different exercise modalities across specific functional recovery domains after AIS. Whole-body tilt training or unilateral strengthening should be preferred for addressing balance deficits. Home-based, technology-assisted functional training should be adopted for ADL recovery. Graded motor imagery combined with electrical stimulation should be applied for motor control. Integrating visual feedback with strength training is effective for improving gait endurance. Sensor-assisted gait training can improve functional mobility. These results offer an evidence-based framework for clinicians to optimize post-stroke rehabilitation protocols and highlight priority areas for future dose–response and long-term outcome research.

## Figures and Tables

**Table 1:** Basic Characteristics of Included Studies.

No.	First Author	Publication Year	Author‘s Country	Source of the Patients	Interventions	Number of Cases	Total Number of Cases	Age	Sex (Male/ Female)	Treatment Course	Outcome Indicators
1	Jiae Kim [13]	2025	Republic of Korea	Single-center	G1: Robot-assisted gait training G2: Gait training	22 22	44	59.2 ± 14.0 64.0 ± 10.2 *	27/17	8 weeks	BBS
2	Liying Zhang [14]	2025	China	Single-center	G1: Robot-assisted Tai Chi training G2: Robot-assisted upper limb training G3: Daily care	29 30 31	90	59.41 ± 11.78 65.44 ± 13.36 63.13 ± 12.51 *	68/28	12 weeks	BI
3	Yong Yu [15]	2025	China	Single-center	G1: Robot-assisted active training G2: Robot-assisted passive training G3: Daily care	15 15 15	45	52.18 ± 14.41 64 ± 9.36 59.21 ± 9.21 *	31/14	2 weeks	FMA
4	Chunli Wan [16]	2025	China	Single-center	G1: Virtual Reality Therapy Combined with Motor Imagery G2: Daily care	14 16	30	57.07 ± 11.65 56.25 ± 15.19 *	24/6	4 weeks	TUG
5	Takuya Hada [17]	2024	Japan	Multi-center	G1: Robot-Assisted Shaking Training G2: Daily care	47 46	93	59 [53,67] 59 [53,68] †	43/50	12 weeks	FMA
6	Yinghua Li [18]	2024	China	Single-center	G1: Robot-Assisted Task-Specific Training G2: Task-Oriented Training	22 22	44	63.7 ± 9.4 63.6 ± 11.4 *	33/7	4 weeks	BI
7	Li Zhang [19]	2024	China	Single-center	G1: Functional Training G2: Daily care	31 31	62	52.00 (11.83) 51.68 (12.95) ‡	49/13	4 weeks	FMA, BBS
8	Yihan Liu [20]	2024	China	Single-center	G1: Ba Duan Jin G2: Medication-Psychological Intervention	50 50	100	57.59 ± 11.46 *	50/50	8 weeks	BI
9	Amer Ghoraz [21]	2024	Spain	Multi-center	G1: Functional Training G2: Daily care	32 31	63	64.22 (10.18) 66.29 (8.60) ‡	41/22	8 weeks	BBS, BI
10	S Amala [22]	2024	India	Single-center	G1: Functional Training G2: Daily care	33 33	66	53.8 ± 6.7 *	38/28	5 days	BBS, BI
11	Linjie Fang [23]	2024	China	Single-center	G1: Augmented Reality Gait Training Combined with Electrical Stimulation G2: Gait Training	18 18	36	58.67 ± 9.91 57.00 ± 12.61 *	26/10	4 weeks	FMA, BBS
12	Osman Çoban [24]	2024	Turkey	Single-center	G1: Robot-assisted horse riding training G2: Daily care	15 15	30	64.5 (11.3) 64.2 (11.8) ‡	13/17	4 weeks	BBS, FMA, TUG
13	Szilvia Kóra [25]	2024	Hungary	Multi-center	G1: Robot-assisted gait training G2: Gait training	15 15	30	64.8 ± 5.89 64.7 ± 7.16 *	22/8	5 weeks	BBS, BI, 6MWT
14	Dušica Simić-Panić [26]	2024	Serbia	Single-center	G1: Visual Aids Training G2: Daily care	59 60	127	65 ± 11.98 67.34 ± 10.86 *	60/59	3 weeks	FMA, BI, BBS, 6MWT, TUG
15	Congcong Huo [27]	2024	China	Single-center	G1: Robot-assisted unilateral gait training G2: Daily care	14 16	30	57.93 ± 11.47 55.25 ± 11.16 *	21/9	4 weeks	BBS
16	Sung-Hoon Kim [28]	2023	Republic of Korea	Single-center	G1: Robot-Assisted Hand Training G2: Daily care	18 18	36	60.5 ± 7.8 61.3 ± 8.4 *	17/19	1 month	FMA, BI
17	Momna Asghar [29]	2023	Pakistan	Single-center	G1: Neuromuscular Control Training G2: Gait Training	30 30	60	54 ± 9.5 53 ± 9.4 *	32/28	6 weeks	BBS
18	Chenlan Shao [30]	2023	China	Single-center	G1: Unilateral Strength Training G2: Daily care	69 70	139	64.56 ± 7.08 65.72 ± 5.95 *	84/43	6 weeks	BBS, 6MWT, BI
19	Jie Shen [31]	2023	China	Single-center	G1: Computer-Assisted Balance Training G2: Balance Training	20 20	40	57.40 ± 7.70 56.10 ± 7.99 *	29/11	8 weeks	BBS
20	Fan Jia [32]	2023	China	Single-center	G1: Graded Motor Imagery combined with Electrical Stimulation G2: Electrical Stimulation G3: Graded Motor Imagery	19 18 19	56	61.89 ± 9.43 55.56 ±11.28 58.42 ± 8.82 *	33/23	4 weeks	FMA BI
21	Sana Batool [33]	2022	Pakistan	Single-center	G1: Visual Feedback Training G2: Daily care	32 32	64	55.63 ± 5.9 54.38 ± 8.8 *	36/28	1 month	BBS, BI
22	Long Zhao [34]	2022	China	Single-center	G1: Robot-assisted hand training with combined electrical stimulation G2: Robot-assisted hand training	30 30	60	59.57 ±10.41 62.87 ±8.40 *	39/21	2 weeks	FMA
23	ATTIYA IRSH [35]	2022	Pakistan	Single-center	G1: Neuromuscular Control Training G2: Functional Training	43 43	86	54.29 ±10.04 55.46 ±8.48 *	48/38	6 weeks	TUG, BBS
24	Pantawit Aphiphaksak [36]	2022	Thailand	Single-center	G1: Balance Training G2: Daily care	16 16	32	59.38 (11.09) 59.38 (10.80) ‡	13/19	4 weeks	BI
25	Yen-Nung Lin [37]	2022	Taiwan, China	Single-center	G1: Robot-assisted gait training G2: Daily care	20 20	40	54.1 ± 8.6 56.5 ± 12.9 *	29/11	3–4 weeks	FMA, BBS
26	Faizan Zaffar Kashoo [38]	2022	Saudi Arabia	Single-center	G1: Motor imagery combined with electrical stimulation G2: Motor imagery	32 32	64	59.9 ± 5.6 58.7 ± 5.7 *	49/15	2 weeks	FMA
27	Wonho Choi [39]	2022	Republic of Korea	Single-center	G1: Cognitive-Motor Integration Training G2: Daily care	15 15	30	62.5 ± 11.3 64.7 ± 14.3 *	13/17	4 weeks	BI
28	Guilin Meng [40]	2022	China	Single-center	G1: Robot-assisted gait training G2: Lower limb training G3: Daily care	62 64 61	187	59.36 ± 1.65 55.26 ± 1.32 60.12 ± 1.73 *	103/84	4 weeks	6MWT
29	Pallavi Harjpal [41]	2022	India	Single-center	G1: Neuromuscular Control Training Combined with Resistance Training G2: Neuromuscular Control Training	20 20	40	51.75 ± 7.06 51.50 ± 8.40 *	23/17	6 weeks	BBS
30	Dorra Rakia Allegue [42]	2022	Canada	Multi-center	G1: Virtual Reality Training G2: Daily care	4 5	11	56.4 (17.3) 57.8 (21.8) ‡	5/4	8 weeks	FMA
31	Xiaoxue Zhai [43]	2021	China	Single-center	G1: Robot-assisted physiotherapy G2: Physiotherapy	10 10	20	61.90 ± 9.62 60.00 ± 6.62 *	18/2	2 weeks	BI
32	Kritika Verma [44]	2021	India	Single-center	G1: Visual Feedback Training G2: Daily care	28 28	56	50.67 ± 11.18 49.16 ± 10.28 *	42/14	6 weeks	BBS
33	Patcharee Kooncumchoo [45]	2021	Thailand	Single-center	G1: Gait Training G2: Functional Training	15 15	30	64.33 ± 7.68 63.53 ± 12.16 *	10/5	8 weeks	FMA
34	Qurat Ul Ain [46]	2021	China, Pakistan	Single-center	G1: Virtual Reality Training G2: Daily care	28 28	56	57.48 ± 5.71 57.65 ± 6.81 *	43/7	6 weeks	FMA
35	Wenjun Jiang [47]	2021	China	Single-center	G1: Neural Inhibition Therapy G2: Cross-Limb Training	20 18 19	57	56.52 ± 13.11 54.28 ± 11.68 55.81 ± 9.61 *	30/30	4 weeks	FMA BI
36	Ditouah Didier Niama Nat [48]	2021	Benin, Belgium	Single-center	G1: Home Rehabilitation Guidance G2: Daily care	31 28	59	51.8 (8.4) 55.1 (8.8) ‡	31/28	8 weeks	FMA
37	Eun Kyu Ji [49]	2021	Republic of Korea	Single-center	G1: Graded Movement Imagery G2: Daily care	21 21	42	61.75 ± 11.59 62.70 ± 11.09 *	19/23	8 weeks	BI
38	Shaomin Chen [50]	2021	China	Single-center	G1: Home Rehabilitation Guidance G2: Daily care	59 62	121	55.41 ± 6.78 56.41 ± 6.13 *	85/36	12 months	FMA, BI
39	Chang-Man An [51]	2021	Republic of Korea	Single-center	G1: Whole-body Tilt Posture Training G2: Balance training	15 15	30	60.5 (6.0) 64.7 (6.9) ‡	21/9	3 weeks	BBS, BI
40	Suparna Gangopadhyay [52]	2021	India	Single-center	G1: Gait Training G2: Daily care	15 15	30	52.07 ± 3.6736 52.40 ± 3.906 *	17/13	4 weeks	TUG, BBS
41	Mandy Yuen [53]	2021	Hong Kong, China	Single-center	G1:Ba Duan Jin G2:Daily care	29 29	58	63.1 ± 10.6 62.0 ± 13.8 *	29/29	16 weeks	TUG, BI
42	Anna Rojek [55]	2020	Poland	Single-center	G1: Robot-assisted gait training G2: Daily care	23 21	44	55–85 (69 ± 8) 59–82 (70 ± 6) §	25/19	4 weeks	BI
43	Hatice İkizler May [54]	2020	Turkey	Single-center	G1: Visual Feedback Training G2: Daily care	21 21	42	57.2 ± 7.6 58.8 ± 9.8 *	25/17	4 weeks	BBS, 6 MWT
44	Mania Sheikh [56]	2020	Iran	Single-center	G1: Functional Training G2: Balance Training	18 18	36	69 (6.31) 68.6 (5.69) ‡	21/15	6 weeks	BBS
45	Hyun Sik Yoon [57]	2020	Republic of Korea	Single-center	G1: Neuromuscular Control Training G2: Balance Training	16 15	31	60.4 ± 14.58 *	17/14	4 weeks	BBS
46	Sae Hoon Chung [58]	2019	Republic of Korea	Single-center	G1: Functional Training G2: Daily care	18 17	35	63.1 ± 11.9 62.5 ± 11.3 *	26/9	3 weeks	BBS, FMA, TUG, 6MWT
47	Lina Bunketorp-K¨all [59]	2019	Sweden	Single-center	G1: Horse riding G2: Daily care	41 41 41	123	62.7 (6.7) 62.6 (6.5) 63.7 (6.7) ‡	69/54	12 weeks	6MWT
48	Hye-Kang PARK [60]	2019	Republic of Korea	Single-center	G1: Balance Training G2: Daily care	14 15	29	56.23 ±13.74 57.13 ± 11.73 *	22/7	4 weeks	BBS, BI
49	Jaeyoung Kim [61]	2019	South Korea	Multi-center	G1: Robot-assisted gait training G2: Daily care	25 23	48	57.7 ± 12.9 60.4 ± 13.2 *	33/15	3 weeks	BI, BBS
50	Guanli Xie [62]	2018	China	Multi-center	G1: Tai Chi G2: Balance Training	120 124	244	60.9 ± 8.7 60.1 ± 8.6 *	182/62	12 weeks	BBS
51	Nari YUN [63]	2018	Republic of Korea	Single-center	G1: Robot-assisted gait training G2: Daily care	18 18	36	63.6 ± 8.3 64.3 ± 8.4 *	19/17	3 weeks	BBS, FMA, BI
52	Dae-Sung Park [64]	2017	Republic of Korea	Single-center	G1: Virtual Reality Training G2: Daily care	10 10	20	62.00 ± 17.14 65.30 ± 10.51 *	10/10	6 weeks	FMA, BBS, TUG
53	Hsin-Chieh Lee [65]	2017	Taiwan, China	Single-center	G1: Virtual Reality-Assisted Balance Training G2: Daily care	26 21	47	59.35 ± 8.95 55.76 ± 9.59 *	34/16	6 weeks	BBS, BI, TUG
54	Taesung In [66]	2016	Republic of Korea	Single-center	G1: Virtual Reality Training G2: Daily care	13 12	25	57.31 ±10.53 54.42 ± 11.44 *	15/10	4 weeks	BBS, TUG
55	Yue Zhang [78]	2016	China	Single-center	G1: Hydrotherapy G2: Daily care	18 18	36	56.3 ± 8.18 54.7 ± 7.59 *	17/19	8 weeks	BI
56	Chueh-Ho Lin [67]	2015	Taiwan, China	Single-center	G1: Bilateral Isometric Strength Training G2: Daily care	16 17	33	52.63 ± 10.49 57.47 ± 10.29 *	5/28	4 weeks	BI, FMA
57	Soo-Yeon Kim [68]	2015	Republic of Korea	Single-center	G1: Robot-Assisted Functional Training G2: Daily care	13 13	26	54.1 ± 14.6 50 ± 16,2 *	19/7	4 weeks	BBS
58	Dal-Yeon Hwang [69]	2015	Republic of Korea	Single-center	G1: Tilt Sensor-Assisted Gait Training with Electrical Stimulation G2: Gait Training	15 15	30	50.00 ± 7.55 49.47 ± 5.01 *	17/13	4 weeks	TUG, BBS
59	Ordahan B [70]	2015	Turkey	Multi-center	G1: Balance Training G2: Daily care	25 25	50	56.7 ± 8.9 57.6 ± 9.4 *	31/19	6 weeks	BBS, TUG
60	Kwanghyun Kim [71]	2014	Republic of Korea	Single-center	G1: Large muscle group Strength Training G2: Daily care	14 14	28	58.2 ±10.3 55.9 ± 10.1 *	28/0	6 weeks	BI
61	Patrizio Sale [72]	2014	Italy	Multi-center	G1: Robot-assisted upper limb training G2: Daily care	26 27	53	67.7 (14.2) 67.7 (14.2) ‡	31/22	6 weeks	FMA
62	Young-hyeon Bae [73]	2014	Republic of Korea	Single-center	G1: Robot-assisted gait training combined with electrical stimulation G2: Robot-assisted gait training	10 10	20	45.4 ± 19.7 52.0 ± 16.1 *	13/7	5 weeks	BBS
63	Threethambal Puckree [74]	2014	South Africa	Single-center	G1: Balance Training G2: Daily care	25 25	50	56.84 ±11.87 61.12 ±7.63 *	26/24	6 months	BBS
64	Birgitta Langhammer [75]	2014	Norway	Single-center	G1: High-Intensity Training G2: Daily care	19 18	37	77.7 ± 8.9 72.3 ± 14.2 *	NR	1 year	BI
65	Carron D. Gordon [76]	2013	Jamaica	Multi-center	G1: Aerobic training G2: Physiotherapy	64 64	128	63.4 ± 9.4 64.9 ± 11.1 *	58/70	12 weeks	BI
66	H Gok [77]	2008	Turkey	Multi-center	G1: Balance Training G2: Daily care	15 15	30	57.4 (8.1) ‡	15/15	4 weeks	FMA

Note: Some studies showed slight differences in the sum of the number of participants in each subgroup and the total sample size, mainly because the original studies used the initial full analysis set (ITT) for recording baseline characteristics and the per-protocol set (PP) after excluding loss to follow-up for analyzing the efficacy. All data in the table were extracted directly from the original studies. Age is reported as * mean ± SD, † median [IQR], ‡ mean (SD), or § range (mean ± SD).

## Data Availability

The original contributions presented in the study are included in the article/Supplementary Materials.

## Supplementary Materials

The following supporting information can be downloaded at: https://www.mdpi.com/article/10.3390/life16081240/s1.
