# Supplementary material for: Comparative Efficacy of Exercise Modalities for Motor Function Recovery After Acute Ischemic Stroke: A Systematic Review and Bayesian Network Meta-Analysis
Source: Life (Basel). 2026 Jul 27;16(8):1240. doi: 10.3390/life16081240 (PMC13514623; doi:10.3390/life16081240)
Supplement: Supplementary file 1 [file life-16-01240-s001.zip › Supplementary Figures R1.pdf]

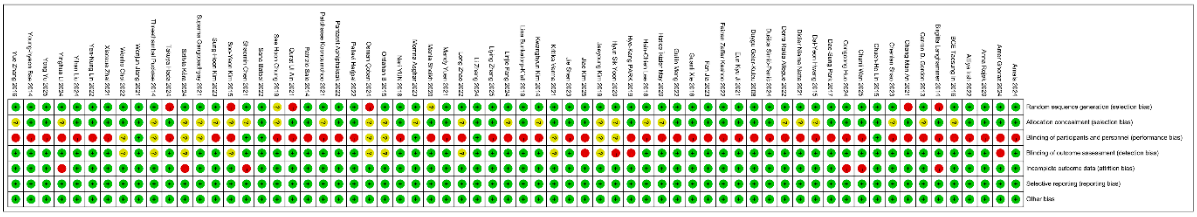

Supplementary Figure S1 Detailed risk of bias assessment for included studies

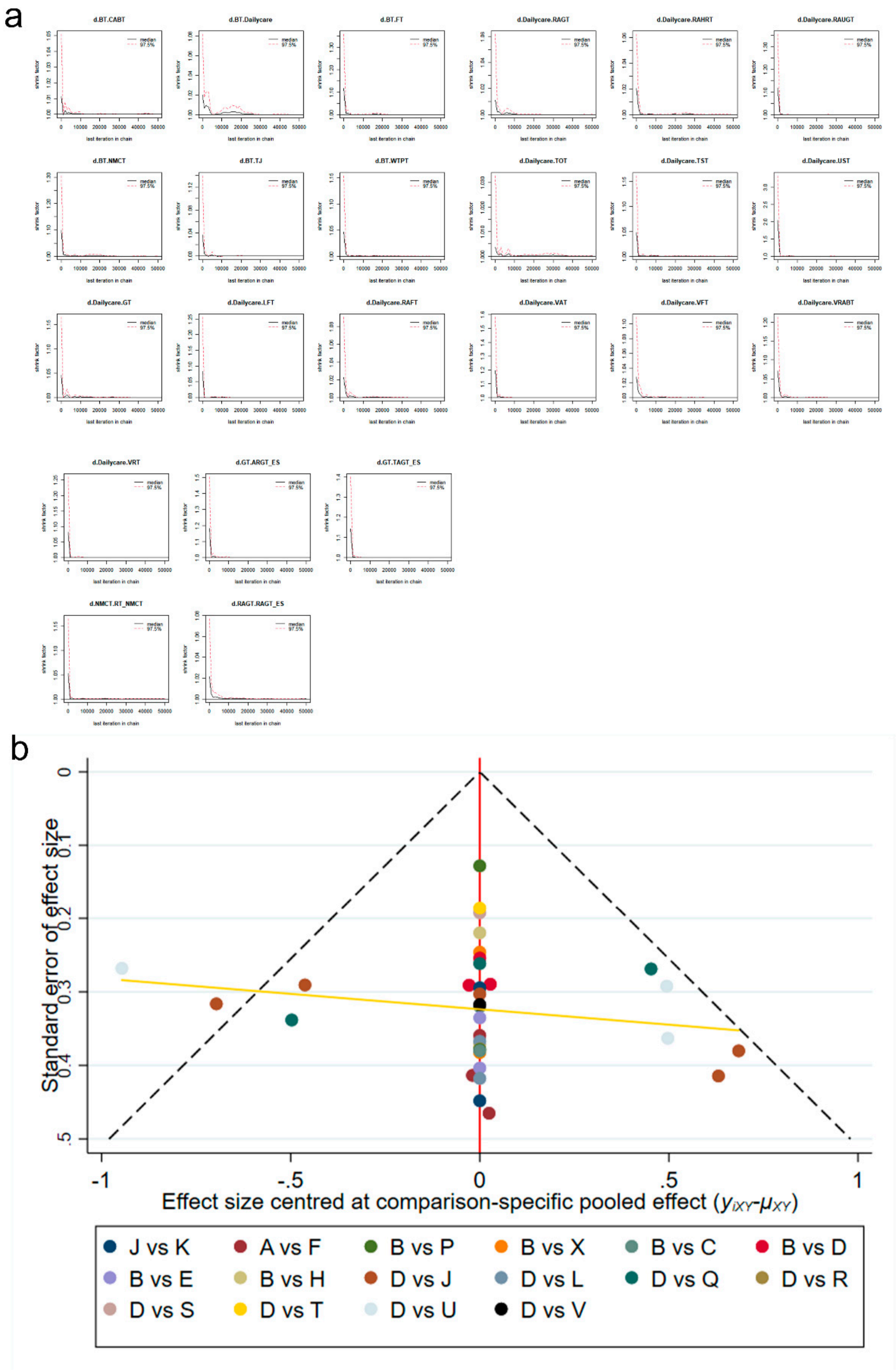

Supplementary Figure S2 (a) Gelman-Rubin diagnostic plots for convergence assessment of the Bayesian network meta-analysis on Berg Balance Scale; (b) Funnel plot for assessing small-study effects in the network meta-analysis on Berg Balance Scale

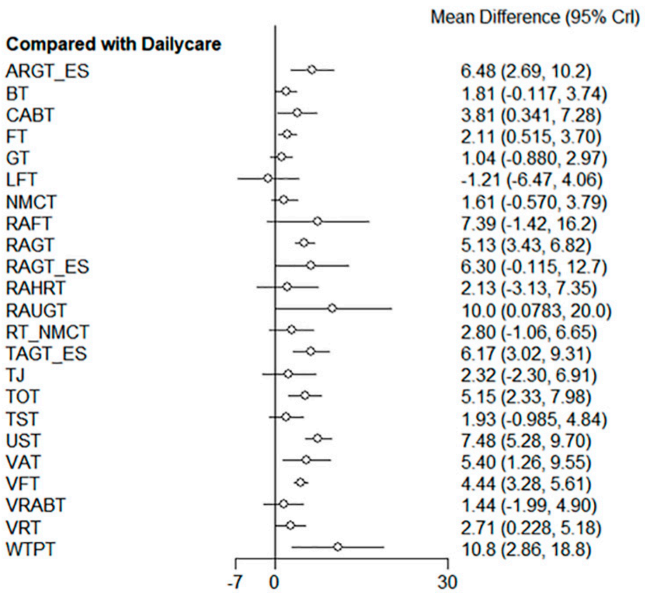

Supplementary Figure S3 Forest plot for the network meta-analysis of the efficacy of different exercise regimens on Berg Balance Scale scores during the rehabilitation phase of acute ischemic stroke

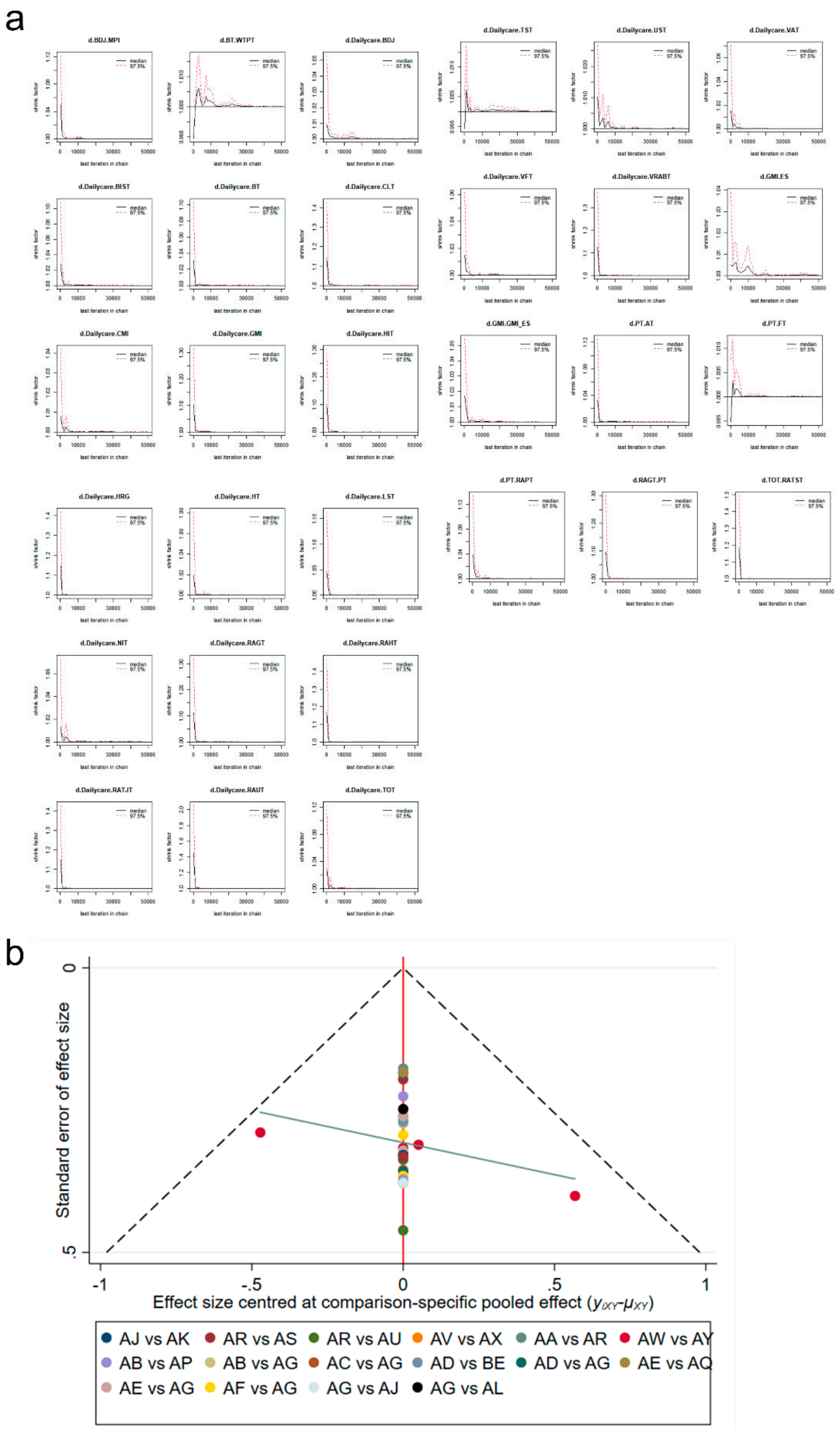

Supplementary Figure S4 (a) Gelman-Rubin diagnostic plots for convergence assessment of the Bayesian network meta-analysis on Barthel Index; (b) Funnel plot for assessing small-study effects in the network meta-analysis on Barthel Index

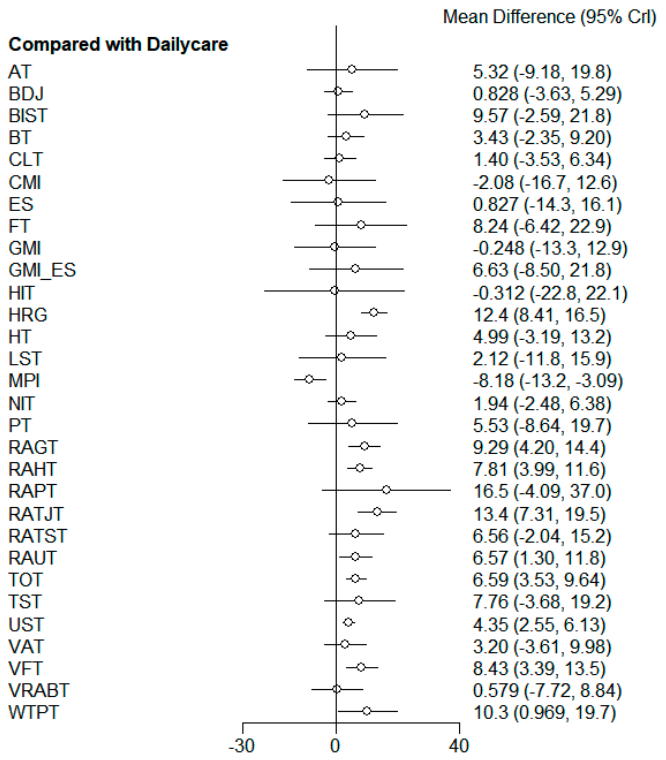

Supplementary Figure S5 Forest plot for the network meta-analysis of the efficacy of different exercise regimens on Barthel Index scores during the rehabilitation phase of acute ischemic stroke

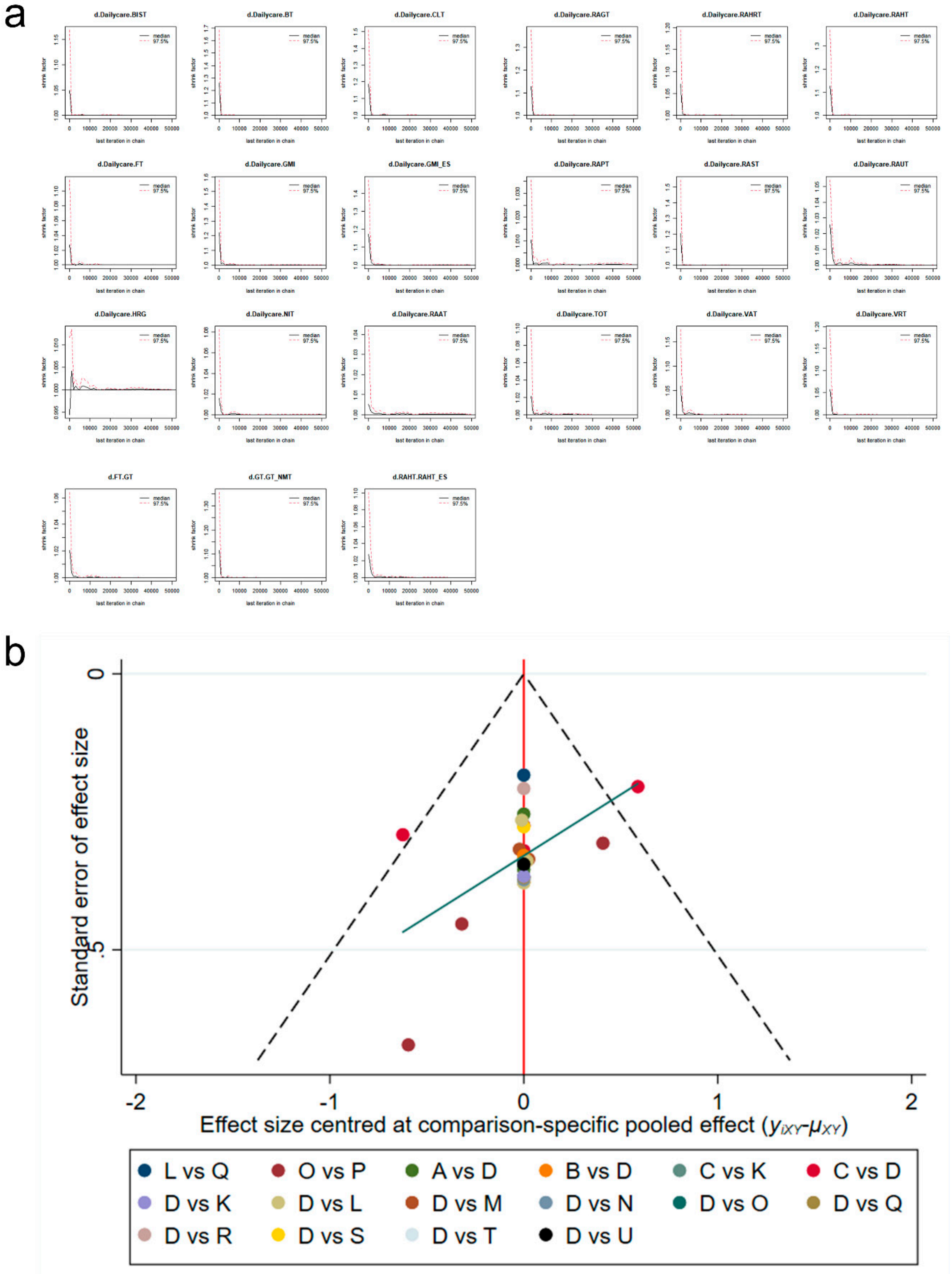

Supplementary Figure S6 (a) Gelman-Rubin diagnostic plots for convergence assessment of the Bayesian network meta-analysis on Fugl-Meyer Assessment; (b) Funnel plot for assessing small-study effects in the network meta-analysis on Fugl-Meyer Assessment

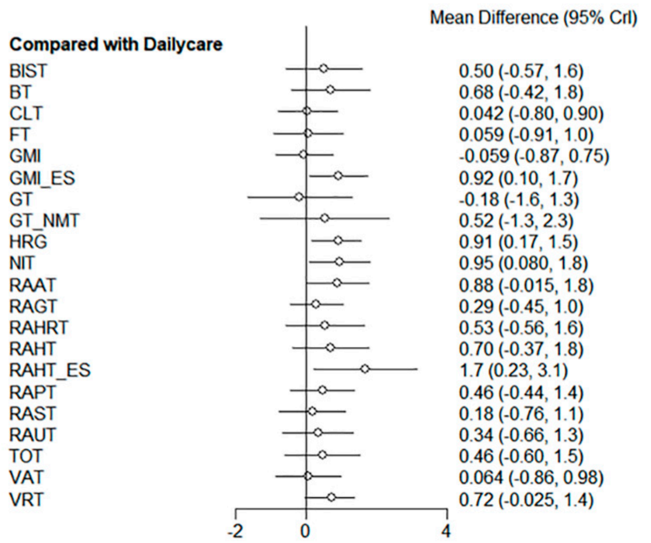

Supplementary Figure S7 Forest plot for the network meta-analysis of the efficacy of different exercise regimens on Fugl-Meyer Assessment scores during the rehabilitation phase of acute ischemic stroke

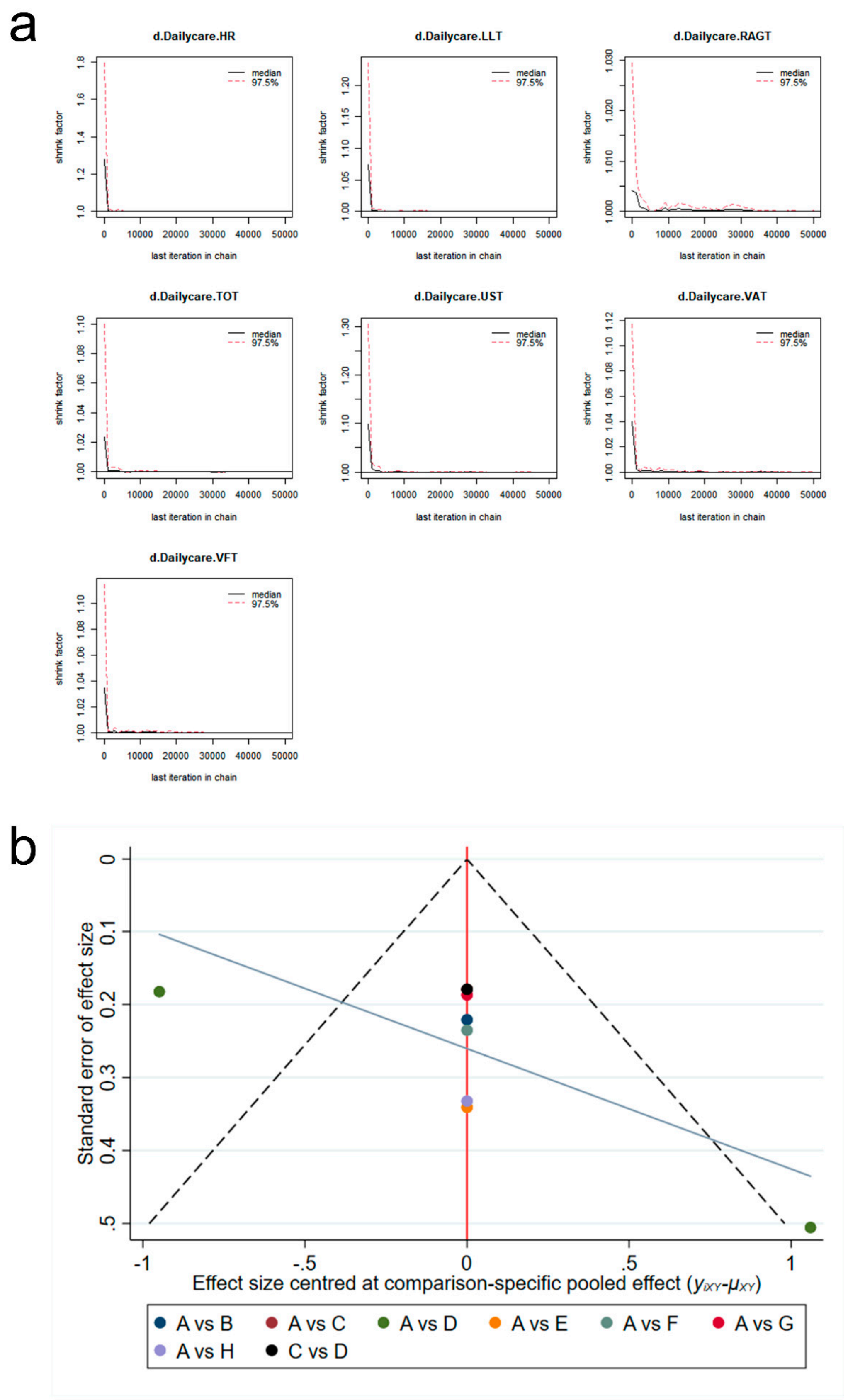

Supplementary Figure S8 (a) Gelman-Rubin diagnostic plots for convergence assessment of the Bayesian network meta-analysis on 6-Minute Walk Test; (b) Funnel plot for assessing small-study effects in the network meta-analysis on 6-Minute Walk Test

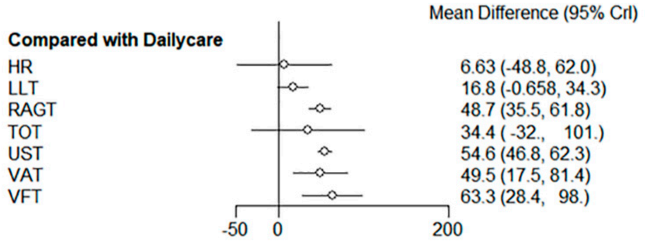

Supplementary Figure S9 Forest plot for the network meta-analysis of the efficacy of different exercise regimens on 6-Minute Walk Test distance during the rehabilitation phase of acute ischemic stroke

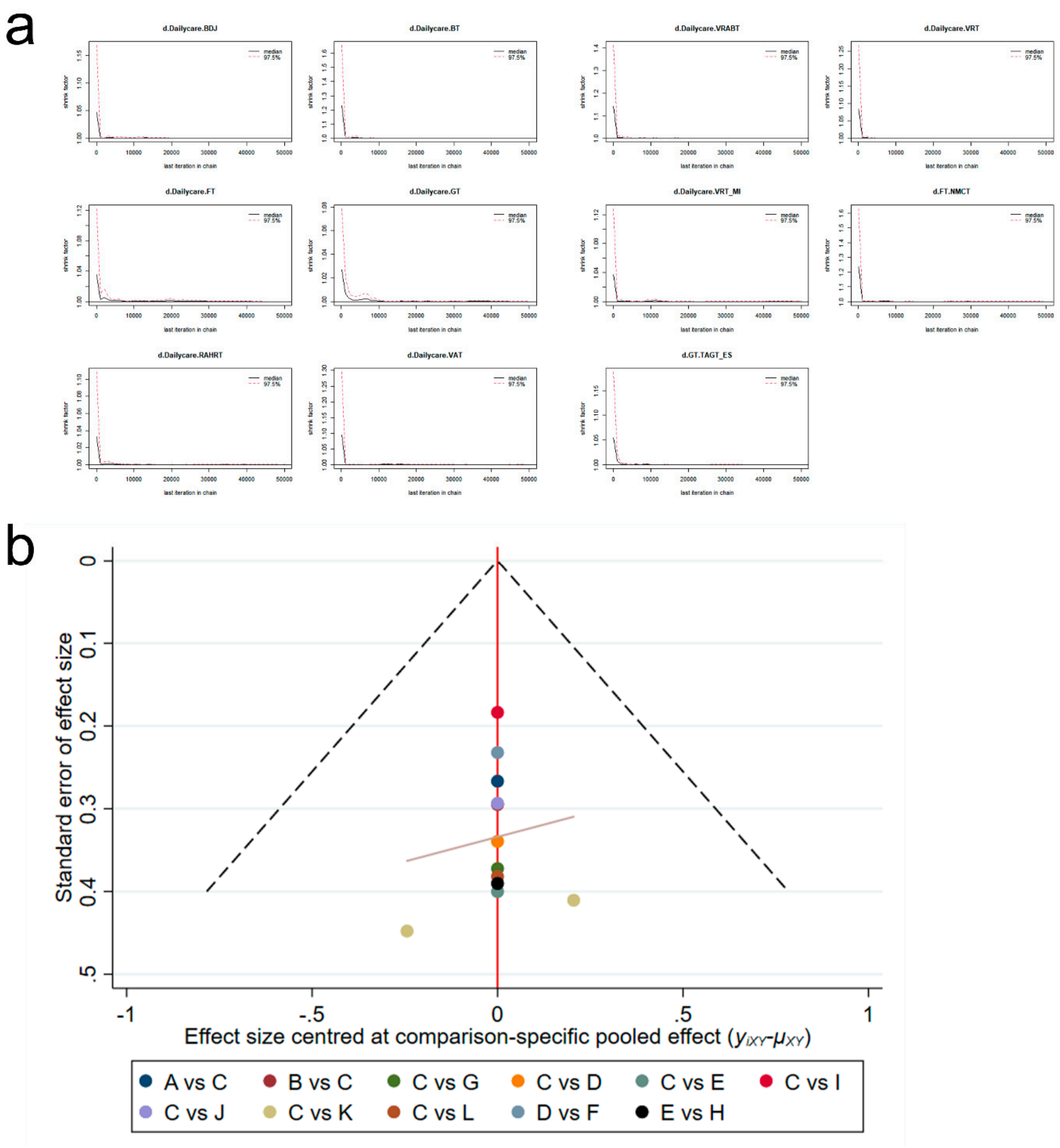

Supplementary Figure S10 (a) Gelman-Rubin diagnostic plots for convergence assessment of the Bayesian network meta-analysis on Timed Up and Go Test scores; (b) Funnel plot for assessing small-study effects in the network meta-analysis on Timed Up and Go Test scores

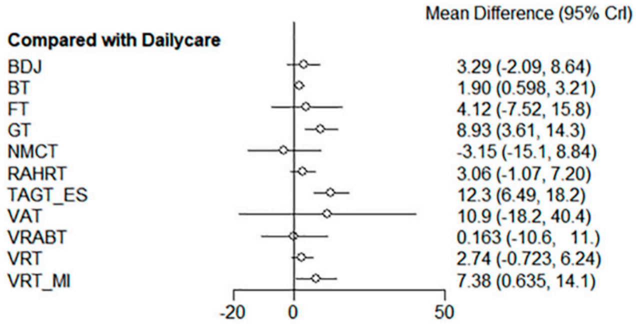

Supplementary Figure S11 Forest plot for the network meta-analysis of the efficacy of different exercise regimens on Timed Up and Go Test time during the rehabilitation phase of acute ischemic stroke
