# Supplementary material for: Comparative Efficacy of Exercise Modalities for Motor Function Recovery After Acute Ischemic Stroke: A Systematic Review and Bayesian Network Meta-Analysis
Source: Life (Basel). 2026 Jul 27;16(8):1240. doi: 10.3390/life16081240 (PMC13514623; doi:10.3390/life16081240)
Supplement: Supplementary file 1 [file life-16-01240-s001.zip › Supplementary Material S2.pdf]

Supplementary Materials S2 Search strategies

1.Pubmed

Search number	Query
#1	"Strokes"[Title/Abstract] OR "Cerebrovascular Accident"[Title/Abstract] OR "Cerebrovascular Accidents"[Title/Abstract] OR "Cerebrovascular Apoplexy"[Title/Abstract] OR "Brain Vascular Accident"[Title/Abstract] OR "Brain Vascular Accidents"[Title/Abstract] OR "Apoplexy"[Title/Abstract] OR "Brain Infarctions"[Title/Abstract] OR "Brain Infarct"[Title/Abstract] OR "Brain Infarcts"[Title/Abstract] OR "Brain Infarction"[Title/Abstract] OR "Brain Venous Infarction"[Title/Abstract] OR "Brain Venous Infarctions"[Title/Abstract] OR "Venous Brain Infarction"[Title/Abstract] OR "Venous Brain Infarctions"[Title/Abstract] OR "Cerebral Stroke"[Title/Abstract] OR "Cerebral Strokes"[Title/Abstract] OR "Stroke, Cerebral"[Title/Abstract] OR "Strokes, Cerebral"[Title/Abstract] OR "Stroke, Acute"[Title/Abstract] OR "Acute Stroke"[Title/Abstract] OR "Acute Strokes"[Title/Abstract] OR "Strokes, Acute"[Title/Abstract] OR "Cerebrovascular Accident, Acute"[Title/Abstract] OR "Acute Cerebrovascular Accident"[Title/Abstract] OR "Acute Cerebrovascular Accidents"[Title/Abstract] OR "Cerebrovascular Accidents, Acute"[Title/Abstract] OR "CVA (Cerebrovascular Accident)"[Title/Abstract] OR "CVAs (Cerebrovascular Accident)"[Title/Abstract] OR "Vascular Accidents, Brain"[Title/Abstract] OR "Cerebrovascular Stroke"[Title/Abstract] OR "Cerebrovascular Strokes"[Title/Abstract] OR "Stroke, Cerebrovascular"[Title/Abstract] OR "Strokes, Cerebrovascular"[Title/Abstract] OR "Apoplexy, Cerebrovascular"[Title/Abstract] OR "Vascular Accident, Brain"[Title/Abstract]
#2	Stroke[MeSH Terms]
#3	"Exercises"[Title/Abstract] OR "Physical Activity"[Title/Abstract] OR "Activities, Physical"[Title/Abstract] OR "Activity, Physical"[Title/Abstract] OR "Physical Activities"[Title/Abstract] OR "Walking"[Title/Abstract] OR "Jogging"[Title/Abstract] OR "Running"[Title/Abstract] OR "swimming "[Title/Abstract] OR "cycling"[Title/Abstract] OR "Active Stretching"[Title/Abstract] OR "Passive Stretching"[Title/Abstract] OR "Relaxed Stretching"[Title/Abstract] OR "Static Stretching"[Title/Abstract] OR "Isometric Stretching"[Title/Abstract] OR "Dynamic Stretching"[Title/Abstract] OR "Ballistic Stretching"[Title/Abstract] OR "Endurance Training"[Title/Abstract] OR "High-Intensity Interval Training"[Title/Abstract] OR "High Intensity Interval Training"[Title/Abstract] OR "High-Intensity Interval Trainings"[Title/Abstract] OR "Sprint Interval Training"[Title/Abstract] OR "Sprint Interval Trainings"[Title/Abstract] OR "Exercise, Physical"[Title/Abstract] OR "Exercises, Physical"[Title/Abstract] OR "Physical Exercise"[Title/Abstract] OR "Physical Exercises"[Title/Abstract] OR "Acute Exercise"[Title/Abstract] OR "Acute Exercises"[Title/Abstract] OR "Exercise, Acute"[Title/Abstract] OR "Exercises, Acute"[Title/Abstract] OR "Exercise, Isometric"[Title/Abstract] OR "Exercises, Isometric"[Title/Abstract] OR "Isometric Exercises"[Title/Abstract] OR "Isometric Exercise"[Title/Abstract] OR "Exercise, Aerobic"[Title/Abstract] OR "Aerobic Exercise"[Title/Abstract] OR "Aerobic Exercises"[Title/Abstract] OR "Exercises, Aerobic"[Title/Abstract] OR "Exercise Training"[Title/Abstract] OR "Exercise Trainings"[Title/Abstract] OR "Training, Exercise"[Title/Abstract] OR "Trainings, Exercise"[Title/Abstract] OR "Resistance Training"[Title/Abstract] OR "Training,

	Resistance"[Title/Abstract] OR "Strength Training"[Title/Abstract] OR "Training, Strength"[Title/Abstract] OR "Weight-Lifting Strengthening Program"[Title/Abstract] OR "Strengthening Program, Weight-Lifting"[Title/Abstract] OR "Strengthening Programs, Weight-Lifting"[Title/Abstract] OR "Weight Lifting Strengthening Program"[Title/Abstract] OR "Weight-Lifting Strengthening Programs"[Title/Abstract] OR "Weight-Lifting Exercise Program"[Title/Abstract] OR "Exercise Program, Weight-Lifting"[Title/Abstract] OR "Exercise Programs, Weight-Lifting"[Title/Abstract] OR "Weight Lifting Exercise Program"[Title/Abstract] OR "Weight-Lifting Exercise Programs"[Title/Abstract] OR "Weight-Bearing Strengthening Program"[Title/Abstract] OR "Strengthening Program, Weight-Bearing"[Title/Abstract] OR "Strengthening Programs, Weight-Bearing"[Title/Abstract] OR "Weight Bearing Strengthening Program"[Title/Abstract] OR "Weight-Bearing Strengthening Programs"[Title/Abstract] OR "Weight-Bearing Exercise Program"[Title/Abstract] OR "Exercise Program, Weight-Bearing"[Title/Abstract] OR "Exercise Programs, Weight-Bearing"[Title/Abstract] OR "Weight Bearing Exercise Program"[Title/Abstract] OR "Weight-Bearing Exercise Programs"[Title/Abstract] OR "free weights"[Title/Abstract] OR "weight machines"[Title/Abstract] OR "elastic bands"[Title/Abstract] OR "body weight exercises"[Title/Abstract] OR "Mind-body exercise"[Title/Abstract] OR "physical and cognitive training"[Title/Abstract] OR "meditation"[Title/Abstract] OR "Relaxations"[Title/Abstract] OR "progressive relaxation"[Title/Abstract] OR "flexibility training"[Title/Abstract] OR "balance training"[Title/Abstract] OR "yoga"[Title/Abstract] OR "Tai ji"[Title/Abstract] OR "tai chi"[Title/Abstract] OR "Taijiquan"[Title/Abstract] OR "Qigong"[Title/Abstract] OR "Ch'i Kung"[Title/Abstract] OR "eight brocade section"[Title/Abstract] OR "eight brocades section"[Title/Abstract] OR "eight-section brocades"[Title/Abstract] OR "eight-section brocades"[Title/Abstract]
#4	Exercise[MeSH Terms]
#5	(#1 OR #2) AND (#3 OR #4)

2.Cochrane

Search number	Query
#1	(‘Strokes’ OR ‘Cerebrovascular Accident’ OR ‘Cerebrovascular Accidents’ OR ‘Cerebrovascular Apoplexy’ OR ‘Brain Vascular Accident’ OR ‘Brain Vascular Accidents’ OR ‘Apoplexy’ OR ‘Brain Infarctions’ OR ‘Brain Infarct’ OR ‘Brain Infarcts’ OR ‘Brain Infarction’ OR ‘Brain Venous Infarction’ OR ‘Brain Venous Infarctions’ OR ‘Venous Brain Infarction’ OR ‘Venous Brain Infarctions’ OR ‘Cerebral Stroke’ OR ‘Cerebral Strokes’ OR ‘Stroke, Cerebral’ OR ‘Strokes, Cerebral’ OR ‘Stroke, Acute’ OR ‘Acute Stroke’ OR ‘Acute Strokes’ OR ‘Strokes, Acute’ OR ‘Cerebrovascular Accident, Acute’ OR ‘Acute Cerebrovascular Accident’ OR ‘Acute Cerebrovascular Accidents’ OR ‘Cerebrovascular Accidents, Acute’ OR ‘CVA (Cerebrovascular Accident)’ OR ‘CVAs (Cerebrovascular Accident)’ OR ‘Vascular Accidents, Brain’ OR ‘Cerebrovascular Stroke’ OR ‘Cerebrovascular Strokes’ OR ‘Stroke, Cerebrovascular’ OR ‘Strokes, Cerebrovascular’ OR ‘Apoplexy, Cerebrovascular’ OR ‘Vascular Accident, Brain’):ti,ab,kw
#2	MeSH descriptor: [Exercise] explode all trees

#3	('Exercises' OR 'Physical Activity' OR 'Activities, Physical' OR 'Activity, Physical' OR 'Physical Activities' OR 'Walking' OR 'Jogging' OR 'Running' OR 'swimming ' OR 'cycling' OR 'Active Stretching' OR 'Passive Stretching' OR 'Relaxed Stretching' OR 'Static Stretching' OR 'Isometric Stretching' OR 'Dynamic Stretching' OR 'Ballistic Stretching' OR 'Endurance Training' OR 'High-Intensity Interval Training' OR 'High Intensity Interval Training' OR 'High-Intensity Interval Trainings' OR 'Sprint Interval Training' OR 'Sprint Interval Trainings' OR 'Exercise, Physical' OR 'Exercises, Physical' OR 'Physical Exercise' OR 'Physical Exercises' OR 'Acute Exercise' OR 'Acute Exercises' OR 'Exercise, Acute' OR 'Exercises, Acute' OR 'Exercise, Isometric' OR 'Exercises, Isometric' OR 'Isometric Exercises' OR 'Isometric Exercise' OR 'Exercise, Aerobic' OR 'Aerobic Exercise' OR 'Aerobic Exercises' OR 'Exercises, Aerobic' OR 'Exercise Training' OR 'Exercise Trainings' OR 'Training, Exercise' OR 'Trainings, Exercise' OR 'Resistance Training' OR 'Training, Resistance' OR 'Strength Training' OR 'Training, Strength' OR 'Weight-Lifting Strengthening Program' OR 'Strengthening Program, Weight-Lifting' OR 'Strengthening Programs, Weight-Lifting' OR 'Weight Lifting Strengthening Program' OR 'Weight-Lifting Strengthening Programs' OR 'Weight-Lifting Exercise Program' OR 'Exercise Program, Weight-Lifting' OR 'Exercise Programs, Weight-Lifting' OR 'Weight Lifting Exercise Program' OR 'Weight-Lifting Exercise Programs' OR 'Weight-Bearing Strengthening Program' OR 'Strengthening Program, Weight-Bearing' OR 'Strengthening Programs, Weight-Bearing' OR 'Weight Bearing Strengthening Program' OR 'Weight-Bearing Strengthening Programs' OR 'Weight-Bearing Exercise Program' OR 'Exercise Program, Weight-Bearing' OR 'Exercise Programs, Weight-Bearing' OR 'Weight Bearing Exercise Program' OR 'Weight-Bearing Exercise Programs' OR 'free weights' OR 'weight machines' OR 'elastic bands' OR 'body weight exercises' OR 'Mind-body exercise' OR 'physical and cognitive training' OR 'meditation' OR 'Relaxations' OR 'progressive relaxation' OR 'flexibility training' OR 'balance training' OR 'yoga' OR 'Tai ji' OR 'tai chi' OR 'Taijiquan' OR 'Qigong' OR 'eight brocade section' OR 'eight brocades section' OR 'eight-section brocades' OR 'eight-section brocades'):ti,ab,kw
#4	MeSH descriptor: [Stroke] explode all trees
#5	(#1 OR #4) AND (#2 OR #3)
#6	('randomised' OR 'randomized' OR 'Phase II' OR 'Phase III' OR 'Phase IV' OR 'Phase 3' OR 'Phase 2' OR 'Phase 4' OR 'Double-blind' OR 'blind' OR 'multicentre' OR 'placebo-controlled' OR 'placebo'):ti,ab,kw
#7	#5 AND #6

3.Embase

Search number	Query
#1	'strokes':ti,ab,kw OR 'cerebrovascular accident':ti,ab,kw OR 'cerebrovascular accidents':ti,ab,kw OR 'cerebrovascular apoplexy':ti,ab,kw OR 'brain vascular accident':ti,ab,kw OR 'brain vascular accidents':ti,ab,kw OR 'apoplexy':ti,ab,kw OR 'brain infarctions':ti,ab,kw OR 'brain infarct':ti,ab,kw OR 'brain infarcts':ti,ab,kw OR 'brain infarction':ti,ab,kw OR 'brain venous infarction':ti,ab,kw OR 'brain venous infarctions':ti,ab,kw OR 'venous brain infarction':ti,ab,kw OR 'venous brain infarctions':ti,ab,kw OR 'cerebral stroke':ti,ab,kw OR 'cerebral strokes':ti,ab,kw OR 'stroke, cerebral':ti,ab,kw OR 'strokes, cerebral':ti,ab,kw OR 'stroke, acute':ti,ab,kw OR 'acute stroke':ti,ab,kw OR 'acute strokes':ti,ab,kw OR 'strokes, acute':ti,ab,kw OR 'cerebrovascular

	accident, acute':ti,ab,kw OR 'acute cerebrovascular accident':ti,ab,kw OR 'acute cerebrovascular accidents':ti,ab,kw OR 'cerebrovascular accidents, acute':ti,ab,kw OR 'cva (cerebrovascular accident)':ti,ab,kw OR 'cvas (cerebrovascular accident)':ti,ab,kw OR 'vascular accidents, brain':ti,ab,kw OR 'cerebrovascular stroke':ti,ab,kw OR 'cerebrovascular strokes':ti,ab,kw OR 'stroke, cerebrovascular':ti,ab,kw OR 'strokes, cerebrovascular':ti,ab,kw OR 'apoplexy, cerebrovascular':ti,ab,kw OR 'vascular accident, brain':ti,ab,kw
#2	'stroke'/exp
#3	'exercises':ti,ab,kw OR 'physical activity':ti,ab,kw OR 'activities, physical':ti,ab,kw OR 'activity, physical':ti,ab,kw OR 'physical activities':ti,ab,kw OR 'walking':ti,ab,kw OR 'jogging':ti,ab,kw OR 'running':ti,ab,kw OR 'swimming':ti,ab,kw OR 'cycling':ti,ab,kw OR 'active stretching':ti,ab,kw OR 'passive stretching':ti,ab,kw OR 'relaxed stretching':ti,ab,kw OR 'static stretching':ti,ab,kw OR 'isometric stretching':ti,ab,kw OR 'dynamic stretching':ti,ab,kw OR 'ballistic stretching':ti,ab,kw OR 'endurance training':ti,ab,kw OR 'high-intensity interval training':ti,ab,kw OR 'high intensity interval training':ti,ab,kw OR 'high-intensity interval trainings':ti,ab,kw OR 'sprint interval training':ti,ab,kw OR 'sprint interval trainings':ti,ab,kw OR 'exercise, physical':ti,ab,kw OR 'exercises, physical':ti,ab,kw OR 'physical exercise':ti,ab,kw OR 'physical exercises':ti,ab,kw OR 'acute exercise':ti,ab,kw OR 'acute exercises':ti,ab,kw OR 'exercise, acute':ti,ab,kw OR 'exercises, acute':ti,ab,kw OR 'exercise, isometric':ti,ab,kw OR 'exercises, isometric':ti,ab,kw OR 'isometric exercises':ti,ab,kw OR 'isometric exercise':ti,ab,kw OR 'exercise, aerobic':ti,ab,kw OR 'aerobic exercise':ti,ab,kw OR 'aerobic exercises':ti,ab,kw OR 'exercises, aerobic':ti,ab,kw OR 'exercise training':ti,ab,kw OR 'exercise trainings':ti,ab,kw OR 'training, exercise':ti,ab,kw OR 'trainings, exercise':ti,ab,kw OR 'resistance training':ti,ab,kw OR 'training, resistance':ti,ab,kw OR 'strength training':ti,ab,kw OR 'training, strength':ti,ab,kw OR 'weight-lifting strengthening program':ti,ab,kw OR 'strengthening program, weight-lifting':ti,ab,kw OR 'strengthening programs, weight-lifting':ti,ab,kw OR 'weight lifting strengthening program':ti,ab,kw OR 'weight-lifting strengthening programs':ti,ab,kw OR 'weight-lifting exercise program':ti,ab,kw OR 'exercise program, weight-lifting':ti,ab,kw OR 'exercise programs, weight-lifting':ti,ab,kw OR 'weight lifting exercise program':ti,ab,kw OR 'weight-lifting exercise programs':ti,ab,kw OR 'weight-bearing strengthening program':ti,ab,kw OR 'strengthening program, weight-bearing':ti,ab,kw OR 'strengthening programs, weight-bearing':ti,ab,kw OR 'weight bearing strengthening program':ti,ab,kw OR 'weight-bearing strengthening programs':ti,ab,kw OR 'weight-bearing exercise program':ti,ab,kw OR 'exercise program, weight-bearing':ti,ab,kw OR 'exercise programs, weight-bearing':ti,ab,kw OR 'weight bearing exercise program':ti,ab,kw OR 'weight-bearing exercise programs':ti,ab,kw OR 'free weights':ti,ab,kw OR 'weight machines':ti,ab,kw OR 'elastic bands':ti,ab,kw OR 'body weight exercises':ti,ab,kw OR 'mind-body exercise':ti,ab,kw OR 'physical and cognitive training':ti,ab,kw OR 'meditation':ti,ab,kw OR 'relaxations':ti,ab,kw OR 'progressive relaxation':ti,ab,kw OR 'flexibility training':ti,ab,kw OR 'balance training':ti,ab,kw OR 'yoga':ti,ab,kw OR 'tai ji':ti,ab,kw OR 'tai chi':ti,ab,kw OR 'taijiquan':ti,ab,kw OR 'qigong':ti,ab,kw OR 'eight brocade section':ti,ab,kw OR 'eight brocades section':ti,ab,kw OR 'eight-section brocades':ti,ab,kw
#4	'exercise'/exp
#5	(#1 OR #2) AND (#3 OR #4)
#6	#5 AND 'randomized controlled trial'/de

4.Web of science

Search number	Query
#1	TS=("Stroke" OR "Strokes" OR "Cerebrovascular Accident" OR "Cerebrovascular Accidents" OR "Cerebrovascular Apoplexy" OR "Brain Vascular Accident" OR "Brain Vascular Accidents" OR "Apoplexy" OR "Brain Infarctions" OR "Brain Infarct" OR "Brain Infarcts" OR "Brain Infarction" OR "Brain Venous Infarction" OR "Brain Venous Infarctions" OR "Venous Brain Infarction" OR "Venous Brain Infarctions" OR "Cerebral Stroke" OR "Cerebral Strokes" OR "Stroke, Cerebral" OR "Strokes, Cerebral" OR "Stroke, Acute" OR "Acute Stroke" OR "Acute Strokes" OR "Strokes, Acute" OR "Cerebrovascular Accident, Acute" OR "Acute Cerebrovascular Accident" OR "Acute Cerebrovascular Accidents" OR "Cerebrovascular Accidents, Acute" OR "CVA (Cerebrovascular Accident)" OR "CVAs (Cerebrovascular Accident)" OR "Vascular Accidents, Brain" OR "Cerebrovascular Stroke" OR "Cerebrovascular Strokes" OR "Stroke, Cerebrovascular" OR "Strokes, Cerebrovascular" OR "Apoplexy, Cerebrovascular" OR "Vascular Accident, Brain")
#2	TS=("Exercise" OR "Exercises" OR "Physical Activity" OR "Activities, Physical" OR "Activity, Physical" OR "Physical Activities" OR "Walking" OR "Jogging" OR "Running" OR "swimming " OR "cycling" OR "Active Stretching" OR "Passive Stretching" OR "Relaxed Stretching" OR "Static Stretching" OR "Isometric Stretching" OR "Dynamic Stretching" OR "Ballistic Stretching" OR "Endurance Training" OR "High-Intensity Interval Training" OR "High Intensity Interval Training" OR "High-Intensity Interval Trainings" OR "Sprint Interval Training" OR "Sprint Interval Trainings" OR "Exercise, Physical" OR "Exercises, Physical" OR "Physical Exercise" OR "Physical Exercises" OR "Acute Exercise" OR "Acute Exercises" OR "Exercise, Acute" OR "Exercises, Acute" OR "Exercise, Isometric" OR "Exercises, Isometric" OR "Isometric Exercises" OR "Isometric Exercise" OR "Exercise, Aerobic" OR "Aerobic Exercise" OR "Aerobic Exercises" OR "Exercises, Aerobic" OR "Exercise Training" OR "Exercise Trainings" OR "Training, Exercise" OR "Trainings, Exercise" OR "Resistance Training" OR "Training, Resistance" OR "Strength Training" OR "Training, Strength" OR "Weight-Lifting Strengthening Program" OR "Strengthening Program, Weight-Lifting" OR "Strengthening Programs, Weight-Lifting" OR "Weight Lifting Strengthening Program" OR "Weight-Lifting Strengthening Programs" OR "Weight-Lifting Exercise Program" OR "Exercise Program, Weight-Lifting" OR "Exercise Programs, Weight-Lifting" OR "Weight Lifting Exercise Program" OR "Weight-Lifting Exercise Programs" OR "Weight-Bearing Strengthening Program" OR "Strengthening Program, Weight-Bearing" OR "Strengthening Programs, Weight-Bearing" OR "Weight Bearing Strengthening Program" OR "Weight-Bearing Strengthening Programs" OR "Weight-Bearing Exercise Program" OR "Exercise Program, Weight-Bearing" OR "Exercise Programs, Weight-Bearing" OR "Weight Bearing Exercise Program" OR "Weight-Bearing Exercise Programs" OR "free weights" OR "weight machines" OR "elastic bands" OR "body weight exercises" OR "Mind-body exercise" OR "physical and cognitive training" OR "meditation" OR "Relaxations" OR "progressive relaxation" OR "flexibility training" OR "balance training" OR "yoga" OR "Tai ji" OR "tai chi" OR "Taijiquan" OR "Qigong" OR "Ch'i Kung" OR "eight brocade section" OR "eight brocades section" OR "eight-section brocades" OR "eight-section brocades")
#3	TS=("randomised" OR "randomized" OR "Phase II" OR "Phase III" OR "Phase IV" OR "Phase 3" OR "Phase 2" OR "Phase 4" OR "Double-blind" OR "blind" OR "placebo-controlled" OR "placebo")

#4	#1 AND #2 AND #3
